# Species Dependent Toxicity Comparison Outcome

**DOI:** 10.1002/advs.202523889

**Published:** 2026-03-31

**Authors:** Lian Xiao, Zhan Yu, Sihang Liu, Yuheng Liu, Chong Deng, Yugang Zhao, Yi Huang, Zhi‐Gang Zheng

**Affiliations:** ^1^ School of Physics East China University of Science and Technology Shanghai China; ^2^ Division of Physics and Applied Physics School of Physical and Mathematical Sciences Nanyang Technological University Singapore Singapore; ^3^ Beijing An Zhen Hospital Affiliated of Capital University of Medical Sciences Beijing China; ^4^ Research Institute of Aero‐Engine Beihang University Beijing China; ^5^ Shanghai Key Laboratory of Multiphase Flow and Heat Transfer in Power Engineering, School of Energy and Power Engineering University of Shanghai for Science and Technology Shanghai China

**Keywords:** biosafe, perovskite, toxicity comparison, toxicity evaluation, toxicity mechanism

## Abstract

Toxicity comparison plays a crucial role in material selection across diverse application fields. Accurate and systematic comparison of toxicity responses among different materials is essential for developing environmentally friendly and safer advanced technologies. In this study, we report a unique phenomenon in toxicity comparison: the relative toxicity ranking between two perovskite compositions reverses when the animal species is changed. We quantitatively assess and compare toxicity responses ranging from macroscopic observations (body weight, organ index, and blood biochemistry) to molecular‐level responses (transcriptomic alterations) across different species and material compositions. Our results reveal that the toxicity variation induced by a species change (from mice to rabbits) exceeds that caused by a compositional change (from lead to tin perovskite). The tiny overlap of damaged genes and biological pathways between mice and rabbits accounts for the pronounced interspecies toxicity differences. Furthermore, tin‐based perovskites display greater sensitivity to species variation than their lead‐based counterparts. Consequently, while lead‐based perovskites are more toxic in mice, this trend reverses in rabbits, where tin‐based perovskites exhibit higher toxicity. This work provides new insight into how species‐dependent biomolecular responses fundamentally shape toxicity comparison outcomes.

## Graphical abstract

By quantitatively assessing and comparing toxicity responses across different animal species and material compositions, our data reveal that toxicity rankings among different materials may be reversed across species.

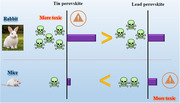

## 1 Introduction

Toxicity comparison plays a pivotal role in guiding the rational selection and safe application of materials across diverse fields, ranging from biomedicine and optoelectronics to environmental engineering [1, 2, 3, 4, 5, 6]. By systematically evaluating and comparing toxicological profiles, researchers can identify candidates that combine optimal performance with minimal adverse effects, thereby ensuring safety in translational and commercial use. Such comparisons not only facilitate compliance with regulatory requirements but also provide mechanistic insights into toxicity pathways [7, 8, 9, 10, 11, 12, 13, 14], enabling the rational design of safer and more sustainable materials. Moreover, conducting side‐by‐side assessments and comparisons under standardized conditions reduce uncertainty and improving reproducibility [15, 16, 17, 18, 19, 20]. Ultimately, rigorous toxicity comparison is essential for advancing eco‐friendly technologies and mitigating potential risks to human health and the environment.

**FIGURE 1:**
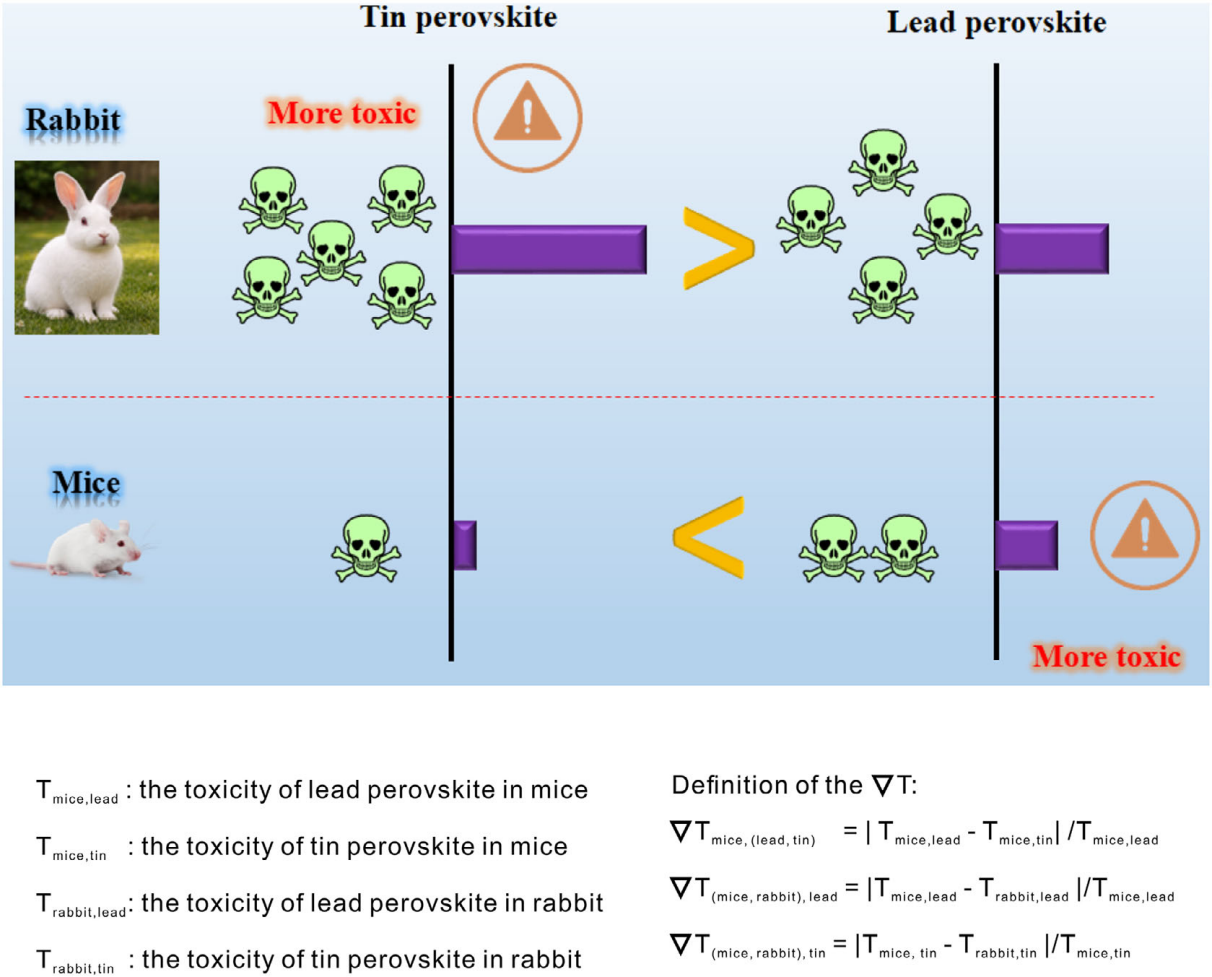
Illustration of the toxicity comparison between lead and tin perovskites in different animal species (mice and rabbits), along with the definition of their toxicities (T) and the corresponding changes (∇ ***T***) observed when varying perovskite composition and animal species.

Generally, the comparative toxicity of two chemicals, A and B, is expected to yield a definitive conclusion—either A is more toxic or B is more hazardous. However, in this work, we reveal an anomalous toxicity comparison phenomenon: the relative toxicity ranking between lead‐ and tin‐based perovskites reverses when the animal species is changed from mice to rabbits. To enable systematic analysis and comparison of toxicity responses across different animal species and material compositions, all toxicity indicators—including body weight, organ index, and blood biochemistry—were normalized to their corresponding control groups. This normalization transforms all data into dimensionless values, thereby allowing quantitative and direct comparison of toxicity responses within a unified framework. Mechanistically, we uncover that the damaged physiological functions induced by perovskites in mice and rabbits kidney show minimal overlap (<1%). Consequently, the variation in toxicity associated with a shift in animal species (from mice to rabbits) is more pronounced than that caused by altering the perovskite composition (from lead to tin). Furthermore, tin‐based perovskites display greater sensitivity to species changes than their lead‐based counterparts. As a result, while lead‐based perovskites are more toxic in mouse, the trend reverses in rabbits, with tin‐based perovskites showing higher toxicity (Figure 1). Our work provides new insights into how species‐dependent biomolecular responses fundamentally shape toxicity comparison outcomes.

## 2 Results and Discussion

### 2.1 Toxicity and Toxicity Change Definition

Perovskite have achieved great success in optoelectronics such as solar cells [21, 22, 23], light‐emitting diodes (LEDs) [24, 25], lasers [26, 27], X‐ray detector [28], and photodetectors [29] while the bio toxicity remains a main concern [11, 12, 13, 30, 31, 32, 33, 34, 35, 36]. Toxicity can be described by multiple parameters, ranging from macroscopic observations in animals (e.g., body weight changes) to molecular‐level responses (e.g., transcriptomic alterations). To enable a quantitative and comprehensive comparison of toxicity across different materials and animal species, we first define toxicity and normalize all evaluation parameters to their corresponding control groups. Specifically, parameters including body weight, organ weight indices, blood biochemistry, and hematological profiles are each normalized relative to the control group. In this framework, the toxicity of lead‐ and tin‐based perovskites in mice are named as *T*_*mice*, *lead*_ and *T*_*mice*, *tin*_ respectively, while *T*_*rabbit*, *lead*_ and *T*_*rabbit*, *tin*_ represent the corresponding values in rabbits. Then the toxicity of the control group, *T_contorl_* is regarded as 1.

To further evaluate and compare the influence of material composition and animal species on toxicity variation, we introduce a dimensionless parameter ∇*T* to quantify toxicity shifts. Since toxicity parameters across different compositions and animal species cannot be directly compared, we propose a new strategy that evaluates the percentage change relative to the corresponding control. This approach enables quantitative comparison of toxicity responses across both species and material compositions. In our study, we selected *T*_*mice*, *lead*_ as the reference group. The toxicity change from lead‐ to tin‐based perovskites in mice is then defined as: ∇ *T_mice_* =  ∇*T*_*mice*, (*lead*,  *tin*)_ = absolute value of |*T*_*mice*, *lead*_ − *T*_*mice*, *tin*_ |/*T*_*mice*, *lead*_. Following the same principle, the species‐dependent toxicity change for lead perovskites (from mice to rabbit) is calculated as: ∇ *T_lead_* =  ∇ *T*_(*mice*, *rabbit*),  *lead*_ = absolute value of |*T*_*mice*, *lead*_ − *T*_*rabbit*, *lead*_ |/*T*_*mice*, *lead*_. Because both ∇*T*_*mice*, (*lead*,  *tin*)_ and ∇*T*_(*mice*, *rabbit*),  *lead*_ are dimensionless quantities that share the same reference group (*T*_*mice*, *lead*_), they allow quantitative comparison of the effects of material composition and animal species on toxicity responses within a unified framework. In our definition system, the choice of reference group is arbitrary; any group can be selected as the reference, and the corresponding calculations for toxicity change follow the same principle. In the main text, we adopt *T*_*mice*, *lead*_ as the reference group for analysis. All definitions are summarized in Figure 1.

### 2.2 Toxicity Comparison in Mice

**FIGURE 2:**
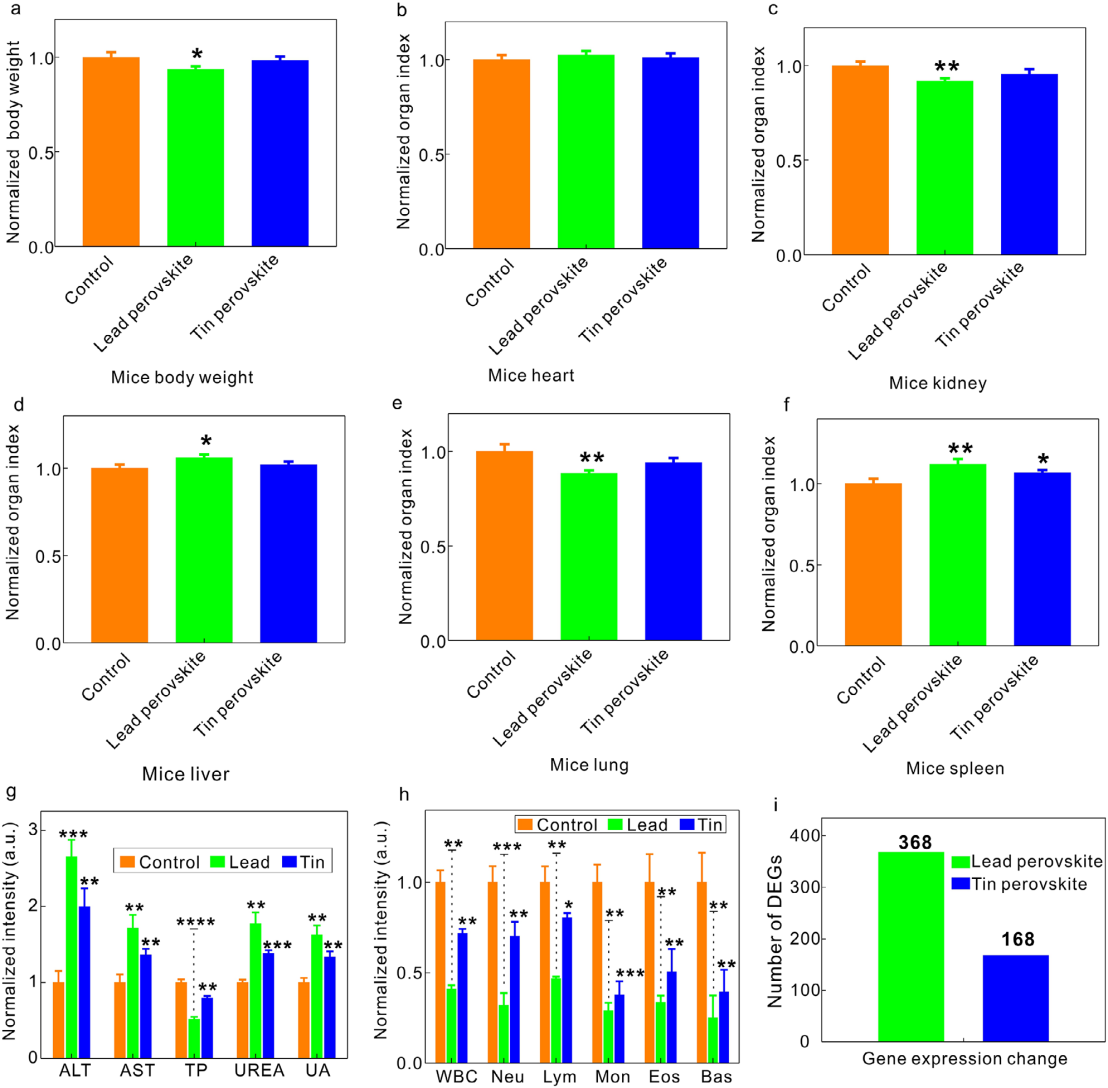
Toxicity comparison of lead and tin perovskite in mice. All toxicity assessment parameters are normalized to the corresponding control group; original data are provided in the Figure S1. (a) normalized mice body weight, (b–f) normalized organ weight index (organ weight/mice body) for mice major organs. (b) heart, (c)kidney, (d)liver, (e)lung, and (f) spleen. (g) Normalized blood biochemistry measurement results in mice. Liver indicators: alanine aminotransferase (ALT), aspartate aminotransferase (AST), and total protein (TP). Kidney indicator: blood urea nitrogen (UREA) and uric acid (UA). (h) Normalized hematology measurement data. Blood immune cells: WBC (white blood cells), Neu (neutrophils), Lym (lymphocytes), Mon (monocytes), Eos (eosinophils), Bas (basophils). (i) The number of differentially expressed genes (DEGs) in lead and tin perovskite treatment groups (kidney tissue). For plots (a–f): *n* = 8, error bars show mean ± SEM; For plots (g,h) *n* = 3 or 4, error bars show mean ± SEM. Statistical significance was calculated using one‐sided Student's t‐test (*p* < 0.05, ^*^ *p* ≤ 0.01, ^**^ *p* ≤ 0.001, ^***^ *p* ≤ 0.0001). Daily intake: 20 mg/kg of lead from lead halide perovskite, or 20 mg/kg of tin from tin halide perovskite.

We start our comparison by examining the biological responses of lead (MAPbI_3_) and tin (MASnI_3_) halide perovskites in mice. Body weight, a key indicator of acute toxicity, was first monitored following oral administration of 20 mg/kg of each perovskite. As shown in Figure 2a, lead perovskite treatment caused a significant decrease in normalized body weight (the original body weight can be seen in Figure S1a), indicating suppressed growth, whereas tin perovskite treatment resulted in body weights comparable to the control group. These results imply that lead halide perovskite has a more pronounced impact on mouse growth than tin halide perovskite under similar exposure conditions. In addition to body weight, we assessed the health status of major organs, including the heart, kidney, liver, lung, and spleen. As shown in Figure 2b, there were no significant differences in the normalized heart weight index (organ weight/body weight) between the control and either perovskite‐treated group. However, notable changes were observed in other organs. Lead perovskite exposure significantly reduced the normalized kidney weight index (Figure 2c), suggesting nephron damage, while the tin perovskite group showed values similar to the control. Lead perovskite‐treated mice also exhibited hepatomegaly (Figure 2d), indicating potential liver disease or metabolic disturbance, whereas the tin perovskite group showed no such change. Given the liver's central role in metabolism and detoxification, this enlargement further underscores the higher toxicity of lead perovskite. In the lungs, lead perovskite exposure caused a significant decrease in normalized lung weight index (Figure 2e), suggesting impaired respiratory function, while the tin perovskite group remained comparable to the control. For the spleen, both perovskite groups exhibited enlargement indicative of inflammation; however, the increase was more pronounced in the lead perovskite group than in the tin perovskite group (Figure 2f).

Given the pivotal roles of the liver and kidney in detoxifying and clearing administered chemicals, we further assessed their function following perovskite exposure using blood biochemistry analysis. Normalized key liver function indicators—alanine aminotransferase (ALT), aspartate aminotransferase (AST), and total protein (TP)—and normalized kidney function markers—blood urea nitrogen (UREA) and uric acid (UA)—are shown in Figure 2g. Both lead and tin perovskite intake elevated ALT and AST levels, enzymes normally confined to the liver; their increased presence in the bloodstream indicates hepatic injury. A reduction in TP further supports liver damage, as the liver is a primary source of blood proteins. Notably, lead perovskite caused a larger increase in ALT and AST and a greater decrease in TP than tin perovskite, suggesting more severe hepatic impairment. Similarly, both perovskite treatments increased UA and UREA levels, indicating impaired clearance and renal dysfunction. Lead perovskite induced a more pronounced elevation of these markers than tin perovskite, reflecting greater kidney impairment. Overall, blood biochemistry results reveal that while both lead and tin perovskites cause liver and kidney damage, the severity is substantially higher for lead perovskite. Beyond assessing major organs, we evaluated the immune system by measuring circulating immune cell counts (normalized)—white blood cells, neutrophils, lymphocytes, monocytes, eosinophils, and basophils (Figure 2h)—which are essential for defending against infections and disease. Halide perovskite exposure reduced the counts of all measured cell types, indicating immune cell damage, with a significantly greater reduction in the lead perovskite group. Specifically, the normalized counts of white blood cells, neutrophils, lymphocytes, monocytes, eosinophils, and basophils decreased to 0.72, 0.70, 0.80, 0.38, 0.51, and 0.39, respectively, in the tin‐perovskite group, and further declined to 0.41, 0.32, 0.47, 0.29, 0.34, and 0.25 in the lead‐perovskite group (Figure 2h). The original data for the organ weight index, kidney & liver function indicators, and immune cell counts are presented in Figure S1b–h.

Beyond physiological assessments, global transcriptional responses were examined by RNA sequencing, with differentially expressed genes (DEGs) analyzed to identify significantly impaired genes (Figure 2i). Lead halide perovskite exposure impaired 2.2‐fold more kidney genes compared to tin halide perovskite. Collectively, analyses of body weight, organ weight indices, blood biochemistry, immune cell counts, and transcriptomic profiles consistently demonstrate that lead halide perovskite is more toxic than tin halide perovskite in mice.

### 2.3 Changes in Toxicity Response Across Animal Species and Material Compositions

**FIGURE 3:**
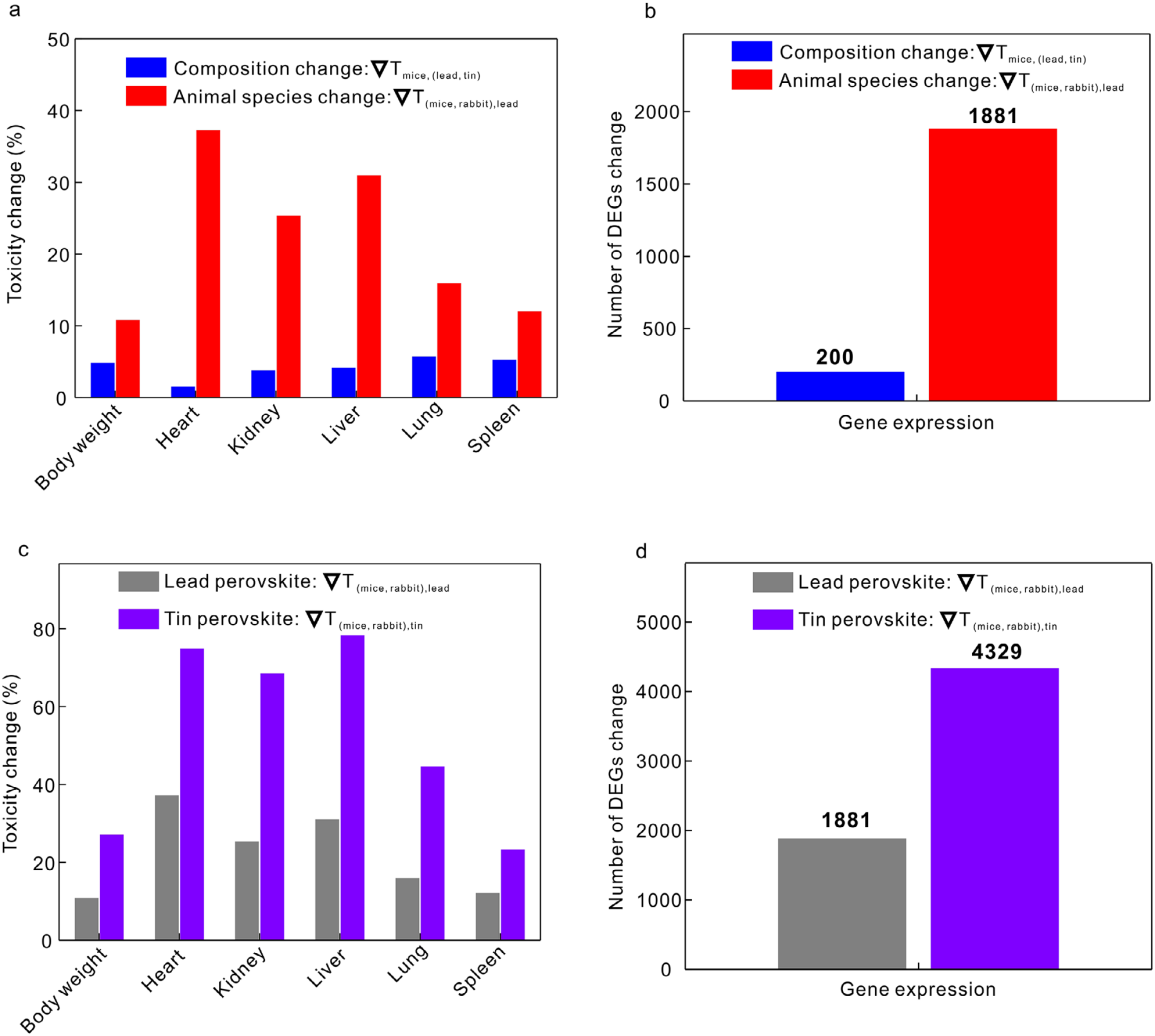
Toxicity changes of perovskites due to composition and species differences. (a,b) Toxicity change from switching composition (lead → tin, ∇ ***T***_***mice***,* *(***lead***, *** tin***)_) and from switching species (mice → rabbits, ∇ ***T***_* *(***mice***, *** rabbit***), ***lead***_). (a) body weight and organ weight indices (heart, kidney, liver, lung, spleen); (b) number of differentially expressed genes (DEGs). The species shift induced a far greater toxicity change than the composition shift. (c,d)The toxicity changes when the animal species is shifted from mice to rabbits for lead (∇ ***T***_* *(***mice***, *** rabbit***), *** lead***_) and tin (∇ ***T***_* *(***mice***, *** rabbit***), *** tin***_) perovskite: (c) The body weight and organ weight index change, (d) number of differentially expressed genes (DEGs) change. Tin perovskite exhibited a more pronounced species‐dependent toxicity change than lead. Daily intake: 20 mg/kg Pb (lead halide perovskite) or 20 mg/kg Sn (tin halide perovskite).

Since both perovskite composition and animal species are involved in toxicity comparison, we need to systematically evaluate their respective contributions to toxicity variation. Because all measurements were normalized to dimensionless values relative to a common reference, quantitative comparison of toxicity responses across species and perovskite compositions become possible. As shown in Figure 3a, shifting the animal species from mice to rabbits for lead perovskite (∇*T*_(*mice*, *rabbit*),  *lead*_) resulted in an 11% change in body weight. In contrast, changing the perovskite composition from lead to tin in mice (∇*T*_*mice*, (*lead*,  *tin*)_) produced only a 5% change. It implies that variation in toxicity resulting from a change in animal species (from mice to rabbits, ∇*T*_(*mice*, *rabbit*),  *lead*_) is greater than that caused by altering the perovskite composition (from lead to tin, ∇*T*_*mice*, (*lead*,  *tin*)_). Similarly, species shifts (with lead perovskite, ∇*T*_ (*mice*, *rabbit*),  *lead*_) induced substantial major organ weight index changes: heart (37%), kidney (25%), liver (31%), lung (16%), and spleen (12%). By comparison, the changes in organ weight index due to perovskite composition change (from lead to tin) in mice (∇*T*_*mice*, (*lead*,  *tin*)_) for heart, kidney, liver, lung and spleen are 2%, 4%, 4%, 6%, and 5% respectively. All organ weight index changes caused by the composition shift from lead to tin (∇*T*_*mice*, (*lead*,  *tin*)_) are smaller than those induced by species shifts with lead perovskite (∇*T*_ (*mice*, *rabbit*),  *lead*_), corresponding to only (∇*T*_*mice*, (*lead*,  *tin*)_/∇*T*_(*mice*, *rabbit*),  *lead*_) 5%, 16%, 13%, 38%, and 42% of the species‐related effects, respectively. Thus, the organ weight index analysis corroborates that the toxicity change induced by altering the animal species (from mice to rabbits) is more pronounced. RNA sequencing (RNA‐seq) analysis further verifies these findings. When the animal species was switched from mice to rabbits (∇*T*_(*mice*, *rabbit*),  *lead*_), we identified 1,881 differentially expressed genes (DEGs) change. By comparison, altering the perovskite composition from lead to tin in mice (∇*T*_*mice*, (*lead*,  *tin*)_) resulted in only 200 DEGs change (Figure 3b). This indicates that the transcriptional response change induced by composition change is much smaller—only 11% of that caused by altering the animal species. Collectively, our data, including body weight, organ weight indices, and RNA‐seq analysis, demonstrate that switching animal species (from mice to rabbits, ∇*T*_ (*mice*, *rabbit*),  *lead*_) exerts a far greater influence on the toxicity response change of halide perovskites than changing the perovskite composition (from lead to tin, ∇*T*_*mice*, (*lead*,  *tin*)_).

We next investigate the different sensitivity of lead and tin perovskite to the animal species change. As depicted in Figure 3c,d, tin perovskite manifests markedly higher sensitivity to animal species alternation than lead perovskite, that is, ∇*T*_ (*mice*, *rabbit*),  *tin*_ is much greater than ∇*T*_(*mice*, *rabbit*),  *lead*_. Specifically, the body weight change induced by switching from mice to rabbits for tin perovskite (∇*T*_ (*mice*, *rabbit*),  *tin*_) is 27%, which is 2.5‐fold higher than that for lead perovskite (11% for ∇*T*_ (*mice*, *rabbit*),  *lead*)_) (Figure 3c). Normalized organ weight index analysis further confirms this trend: upon species shift, tin perovskite causes a change (∇*T*_ (*mice*, *rabbit*),  *tin*_) of 75%, 68%, 78%, 45%, and 23% for the heart, kidney, liver, lung, and spleen, respectively, whereas the corresponding values for lead perovskite (∇*T*_ (*mice*, *rabbit*),  *lead*_) are 37%, 25%, 31%, 16%, and 12% (Figure 3c). Thus, lead perovskite induces only (∇*T*_ (*mice*, *rabbit*),  *lead*_/∇*T*_ (*mice*, *rabbit*),  *tin*_) ∼50%, 37%, 39%, 36%, and 52% of the normalized organ index changes observed for tin perovskite, respectively. RNA‐seq corroborates these findings: the changed DEG count associated with tin perovskite (∇*T*_ (*mice*, *rabbit*),  *tin*_) (4,329) is more than twice that of lead perovskite (1,881 for ∇*T*_ (*mice*, *rabbit*),  *lead*_) upon species shift from mice to rabbits (Figure 3d). Collectively, these results demonstrate that switching from mice to rabbits increases toxicity in both systems, but the effect is substantially more pronounced for tin perovskite, which may critically influence the comparative toxicity outcomes of lead and tin perovskites when the animal species is changed from mice to rabbits.

**FIGURE 4:**
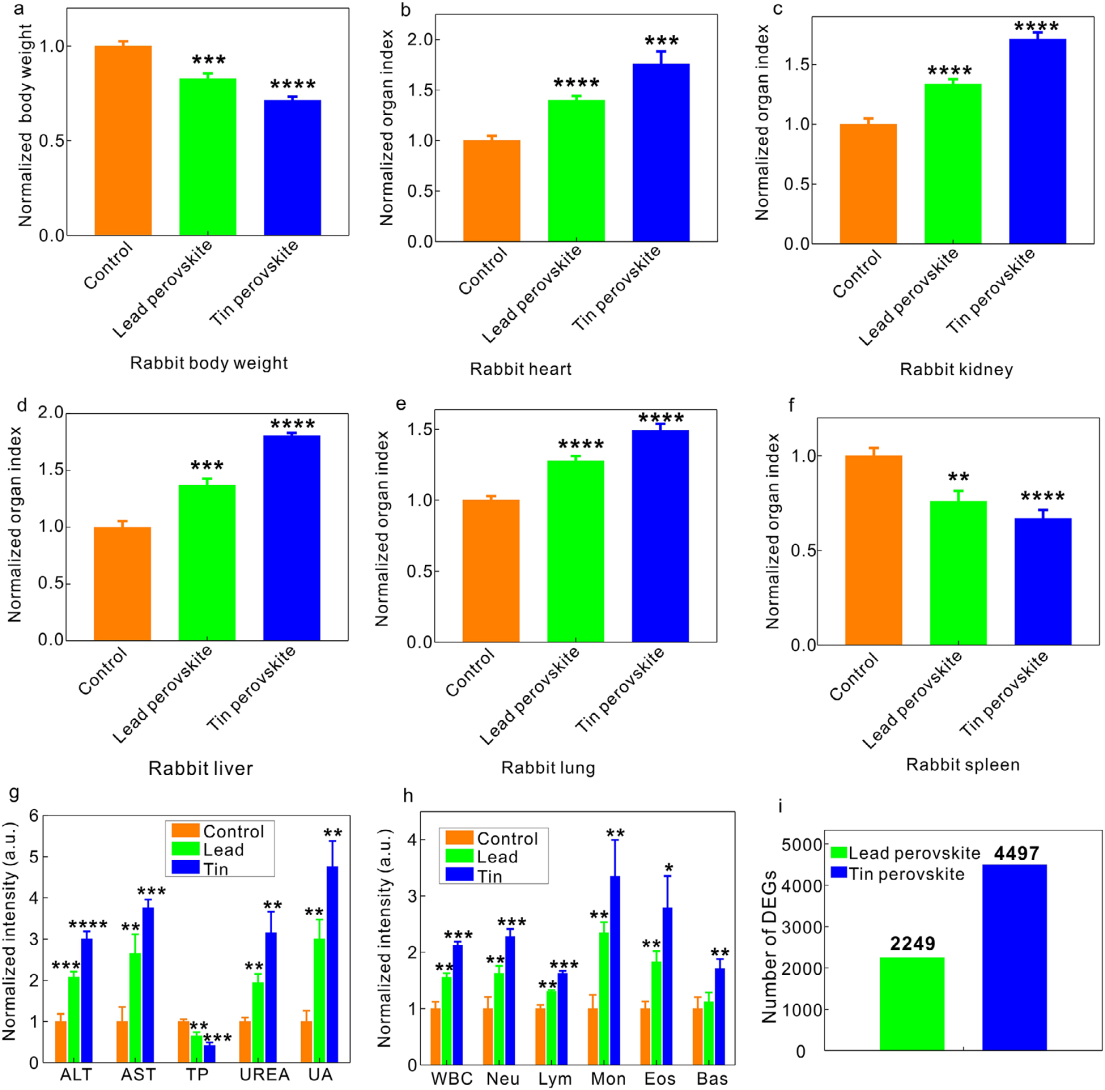
Toxicity comparison of lead and tin perovskite in rabbits. All toxicity assessment parameters are normalized to the corresponding control group. The original data can be seen in Supporting Information. (a) normalized rabbit body weight, (b–f) normalized organ weight index (organ weight/rabbit body) for rabbit major organs. (b) heart, (c)kidney, (d)liver, (e)lung, and (f) spleen. (g) Normalized blood biochemistry measurement results in a rabbit. Liver indicators: alanine aminotransferase (ALT), aspartate aminotransferase (AST), and total protein (TP). Kidney indicator: blood urea nitrogen (UREA) and uric acid (UA). (h) Normalized hematology measurement data. Blood immune cells: WBC (white blood cells), Neu (neutrophils), Lym (lymphocytes), Mon (monocytes), Eos (eosinophils), Bas (basophils). (i) The number of differentially expressed genes (DEGs) in lead and tin perovskite treatment groups (kidney tissue). For plots (a–f): *n* = 8, error bars show mean ± SEM; For plots (g,h): *n* = 8, error bars show mean ± SEM. Significance determined by one‐sided Student's t‐test: *p* < 0.05, ^*^ *p* ≤ 0.01, ^**^ *p* ≤ 0.001, ^***^ *p* ≤ 0.0001. Daily intake: 20 mg/kg Pb (lead halide perovskite) or 20 mg/kg Sn (tin halide perovskite).

Thus, we further study the toxicity comparison of lead and tin perovskite in rabbits. As shown in Figure 4a, the normalized body weight of rabbits exposed to lead and tin perovskites (20 mg/kg daily intake) decreased to 0.83 and 0.71, corresponding to 17% and 29% reductions relative to the control group, respectively, thereby indicating suppressed rabbit growth. Both lead and tin perovskite treatments significantly altered normalized organ weight indices (organ weight/body weight) (Figure 4b–f), with increases in the heart, kidney, liver, and lung indices and a decrease in the spleen index, demonstrating severe organ impairment. In all body weight and organ weight index cases, tin perovskite induced more pronounced changes than lead, confirming its stronger toxicity in rabbits. Normalized blood biochemistry further confirmed liver and kidney impairment. As shown in Figure 4g, both treatments elevated ALT and AST levels while reducing TP, indicating liver damage. Kidney function markers (UREA and UA) were also significantly elevated, suggesting kidney injury. Again, tin perovskite caused greater alterations than lead in all indicators. Blood immune cell analysis revealed a similar trend. Figure 4h shows that exposure to both perovskites increased counts of white blood cells (normalized), neutrophils, lymphocytes, monocytes, eosinophils, and basophils, consistent with systemic inflammation. Tin perovskite produced larger increases across all immune cell types, underscoring its greater toxicity. And the original data for the body weight, organ weight index, kidney & liver function indicators, and immune cell counts of rabbit are presented in SI Figure S2a–h.

Besides, as shown in Figure 4i, tin perovskite exposure induced 4497 differentially expressed genes (DEGs) in kidney, more than twice the number caused by lead perovskite (2249), indicating substantially greater transcriptomic disruption. Taken together, analyses of body weight, organ indices, blood biochemistry, immune cell counts, and gene expression consistently demonstrate that tin perovskite is more toxic than lead perovskite in rabbits. Remarkably, the reversed toxicity comparison between mice and rabbits persists even when the daily intake is reduced from 20 to 1 mg/kg in both species, and the detailed toxicity comparison results at 1 mg/kg are provided in Figures S3 and S4. In real‐world scenarios, the estimated perovskite exposure level is typically in the range of tens to hundreds of µg/kg. Therefore, we additionally evaluated a lower dose of 0.5 mg/kg, which approaches the upper limit of realistic application‐related exposure and the results are shown in Figure S5 and S6. The observed toxicity trends at 0.5 mg/kg were consistent with those obtained at 1 and 20 mg/kg. Nevertheless, under different application scenarios—such as LEDs/lighting, detectors, lasers, or solar cells—the extent of perovskite leakage, environmental release, environmental concentration, and ultimate human intake may vary considerably. Moreover, realistic exposure durations would likely extend over several months or even years, thereby requiring long‐term evaluation. These studies are currently ongoing and will be reported in future work. The dose‐response DEGs curves (kidney tissue) are also shown in Figure S7.

### 2.4 Transcriptional Analysis

**FIGURE 5:**
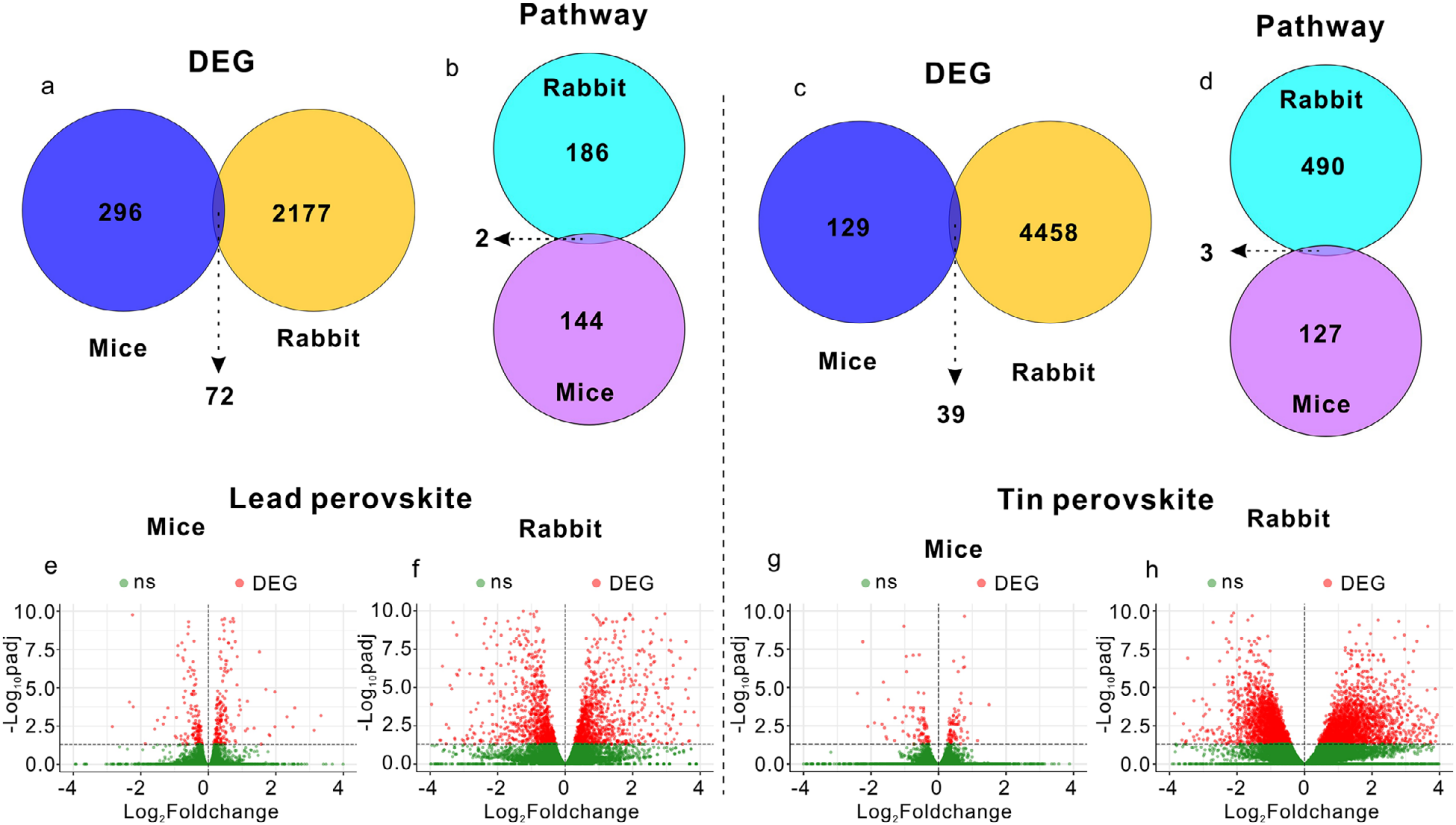
The overlapped damaged genes (differentially expressed genes, DEGs) between mice and rabbit for lead perovskite (a) and tin perovskite (c). Overlapping impaired physiological functions (pathways) between mice and rabbit for (b) lead and (d) tin perovskite. (e,f) DEGs identified by DEseq2 (v1.34.0) for (e) mice (368 DEGs) and (f) rabbits (2249 DEGs) exposed to lead perovskite. (g,h) DEGs identified by DEseq2 (v1.34.0) for (g) mice (168 DEGs) and (h) rabbits (4497 DEGs) exposed to tin perovskite. Daily intake: 20 mg/kg of lead from lead halide perovskite, or 20 mg/kg of tin from tin halide perovskite. All analyses were based on the RNA sequencing data of kidney organs from mice and rabbits.

In this section, we further investigate the mechanisms underlying the pronounced interspecies differences in perovskite toxicity. Since toxicity responses and physiological functions are largely governed by transcriptional regulation and associated pathways, we analyzed the global gene expression changes induced by perovskite exposure in both mice and rabbits. As shown in Figure 5a,e,f, lead perovskite exposure resulted in 368 differentially expressed genes (DEGs) in mice—only 16% of the 2249 DEGs identified in rabbits. Strikingly, only 72 DEGs were shared between the two species, indicating that most of the gene changes induced by lead perovskite in mice are distinct from those in rabbits. This divergence suggests fundamentally different toxicity mechanisms, even under similar exposure conditions. A similar pattern was observed with tin perovskite (Figure 5c,g,h): 168 DEGs were detected in mice and 4497 in rabbits, with only 39 DEGs shared. This highlights species‐specific and largely non‐overlapping toxicity mechanisms. Prior to the physiological analysis, we performed qPCR validation and protein‐level analysis of selected key genes. The corresponding results are presented in Figures S8 and S9 and Tables S1 and S2. The protein expression and qPCR data are consistent with the RNA‐seq results, further supporting the reliability and robustness of the transcriptomic analysis.

To further elucidate the physiological impact, we performed pathway enrichment analysis. Lead perovskite altered 146 biological pathways in mice and 188 in rabbits (Figure 5b), with only two overlapping between the species. Notably, none of the top altered pathways were shared (Figures S10 and S11), reinforcing the conclusion that lead perovskite triggers distinct toxicity mechanisms in the two species. Gene set enrichment analysis (GSEA) of lead perovskite exposed mouse kidney revealed a coordinated and robust transcriptional response that could be consolidated into different functional modules. First, a dominant detoxification and oxidative stress activation module was observed, highlighted by strong enrichment of pathways related to response to toxic substance and detoxification. This module was driven by canonical stress‐responsive genes and ATP‐binding cassette transporters, indicating activation of metal buffering, redox regulation, and xenobiotic clearance mechanisms. Second, a prominent lipid remodeling and cholesterol transport module was enriched, encompassing regulation of lipid localization, cholesterol efflux, sterol transport, and lipase activity pathways. These enriched pathways suggest active metabolic reprogramming and membrane lipid remodeling in response to lead perovskite exposure. Third, a metal and ion homeostasis module was strongly induced, including monoatomic cation homeostasis and intracellular ion balance pathways. The recurrent enrichment of metallothioneins and oxidative enzymes within these gene sets supports a coordinated effort to buffer intracellular metal accumulation and maintain redox equilibrium. Collectively, these findings demonstrate that lead perovskite exposure elicits a transcriptionally driven adaptive response in mouse characterized by detoxification activation, lipid metabolic remodeling, and ion buffering. Similarly, Gene set enrichment analysis (GSEA) of lead perovskite exposed rabbit revealed a striking and coordinated activation of proliferative programs. The top enriched pathways converged into three dominant functional modules: **mitotic cell** cycle activation, chromosome organization and segregation, and DNA replication/genome maintenance. The mitotic module encompassed cell cycle progression, nuclear division, and G2/M transition processes. Concurrently, strong enrichment of chromosome‐ and kinetochore‐associated terms reflected activation of structural segregation machinery and centromere components prominently represented. The DNA metabolic module further demonstrated enrichment of replication and homologous recombination pathways, indicating enhanced DNA synthesis and genome surveillance activity. In addition, enrichment of mitochondrial inner membrane–associated terms suggests bioenergetic remodeling accompanying proliferative activation. Collectively, these data demonstrate that lead perovskite exposure in rabbits induces a robust proliferative and chromosomal activation signature characterized by coordinated upregulation of mitotic machinery, chromosome segregation dynamics, and DNA replication/repair pathways, consistent with heightened cell cycle re‐entry and replication stress–associated remodeling. Comparative analysis of lead perovskite induced transcriptional responses revealed striking species‐specific mechanistic divergence between mouse and rabbit kidney. In mouse, lead perovskite exposure predominantly activated a detoxification and oxidative stress adaptation program, characterized by enrichment of xenobiotic metabolism, metal ion homeostasis, and redox‐regulatory pathways, driven by canonical stress‐response genes including Hmox1, Mt1/2, Nqo1, and glutathione‐related enzymes. This profile indicates engagement of protective buffering mechanisms aimed at limiting metal‐induced oxidative damage. In contrast, the rabbit kidney response was dominated by a robust proliferation–chromosome–replication axis, with strong enrichment of mitotic cell cycle, chromosome segregation, and DNA replication/repair pathways. Leading‐edge genes in rabbit included core regulators of G2/M progression and homologous recombination, such as CDK1, CCNB2, FOXM1, MCM family members, RAD51, and BRCA1, indicating widespread cell cycle re‐entry and activation of genome maintenance machinery. Mechanistically, these findings suggest that while mouse kidney mounts a predominantly cytoprotective detoxification response, rabbit kidney exhibits a pronounced hyperproliferative and replication‐stress–associated program, potentially reflecting differential injury sensing, regenerative dynamics, or species‐specific susceptibility to lead perovskite induced genomic instability. This divergence underscores the importance of cross‐species evaluation when assessing heavy metal nephrotoxicity and mechanistic risk interpretation. The DEG and pathway results were highly consistent: most damaged genes and biological functions were species‐specific. Remarkably, we observed opposite biological responses in mice and rabbit even exposed to the same lead perovskite. For example, lead perovskite exposure upregulated pathways related to metal ion response, zinc ion response, and cellular metal ion homeostasis in mice, but downregulated these same pathways in rabbits (see Figure S14a). Similar results were found with tin perovskite: 130 and 493 pathways were altered in mice and rabbits, respectively (Figure 5d), with only three shared and no overlap among the top 10 altered pathways (Figures S12 and S13). Gene set enrichment analysis (GSEA) of tin perovskite treated mouse revealed a dominant transcriptional activation and proliferative signature. The most significantly enriched term was mitotic cell cycle, indicating robust activation of cell division programs. Consistently, centrosome associated pathways were also significantly enriched, supporting activation of mitotic machinery. Transcriptional regulation pathways were prominently represented among the top‐ranked gene sets. Both regulation of transcription by RNA polymerase II and transcription by RNA polymerase II were significantly enriched, indicating global transcriptional reprogramming. Upstream regulatory terms, including negative regulation of transcription by RNA polymerase II and DNA‐binding transcription factor binding, further support enhanced engagement of transcriptional control networks. In parallel, pathways associated with nucleic acid metabolic process and nucleobase‐containing compound metabolic process were strongly enriched, consistent with increased nucleotide turnover and biosynthetic demand. Collectively, these data indicate that tin perovskite exposure in mouse induces coordinated activation of cell cycle progression, RNA polymerase II–driven transcription, and nucleic acid metabolism, reflecting a proliferative and transcriptionally active state rather than a predominantly metabolic suppression phenotype. Moreover, Gene set enrichment analysis (GSEA) of the tin perovskite treated rabbit revealed pronounced transcriptional reprogramming, with the strongest effects observed in pathways related to membrane structure and metabolic processes. The most significantly altered pathway was G protein–coupled receptor activity (NES = 2.68), indicating robust receptor‐mediated signaling activation. Strikingly, however, the majority of the top‐ranked pathways exhibited negative NES values, reflecting coordinated suppression. These included postsynaptic membrane (NES = −2.58). intracellular protein‐containing complex (NES = −2.55), and synaptic membrane (NES = −2.52), suggesting substantial remodeling of membrane‐associated protein complexes and cellular structural organization. Metabolic pathways were also strongly downregulated, including organic acid catabolic process and carboxylic acid catabolic process (NES = −2.46), as well as fatty acid catabolism and β‐oxidation pathways. These findings indicate suppression of mitochondrial fatty acid oxidation and energy‐producing metabolic programs. In parallel, ATP hydrolysis activity (NES = −2.45) and S‐adenosylmethionine‐dependent methyltransferase activity were also reduced, suggesting attenuation of ATP‐dependent enzymatic and regulatory processes. Collectively, these data reveal that the dominant transcriptional signature in tin perovskite exposed rabbit is characterized by coordinated suppression of metabolic and membrane‐associated pathways, accompanied by receptor‐mediated signaling activation. The magnitude and directionality of these changes suggest a metabolic reprogramming event coupled with structural remodeling, rather than a purely inflammatory‐driven response. Comparative GSEA analysis revealed strikingly divergent transcriptional responses between rabbit and mouse kidneys following tin perovskite exposure. In rabbit, the dominant signature was characterized by strong negative enrichment of metabolic and membrane‐associated pathways, including suppression of fatty acid β‐oxidation, organic acid catabolism, ATP hydrolysis activity, and synaptic/membrane protein complexes. These changes indicate coordinated attenuation of mitochondrial energy metabolism and structural remodeling of membrane‐associated complexes, consistent with a metabolic suppression phenotype. In contrast, mouse exhibited an opposing transcriptional profile dominated by positive enrichment of proliferative and transcriptional programs. The most strongly enriched pathways included mitotic cell cycle, nucleic acid metabolic process, and RNA polymerase II–mediated transcription, accompanied by centrosome activation. This pattern reflects coordinated upregulation of cell cycle progression, transcriptional reprogramming, and nucleotide biosynthesis, consistent with a proliferative and transcriptionally active state. Mechanistically, these findings suggest that tin perovskite exposure elicits fundamentally different biological strategies across species. Rabbit appears to undergo metabolic repression and structural remodeling, indicative of energy conservation or injury‐associated suppression. In contrast, mouse activates a compensatory proliferative response characterized by enhanced transcriptional output and cell cycle engagement. The opposing polarity of enrichment patterns (predominantly negative NES in rabbit versus predominantly positive NES in mouse) underscores species‐specific regulatory programs governing metabolic adaptation and cellular turnover following tin perovskite exposure. Furthermore, 12 pathways—including organelle organization, mitochondrial membrane, and extracellular region etc.—showed opposite regulation in the two species (see Figure S14b).

Finally, we compare the relative species sensitivity to lead and tin perovskites. For lead perovskite, the shared DEGs and pathways between mice and rabbits were 72 and 2, representing only 3% and 1% of the total rabbit responses. In contrast, tin perovskite yielded only 39 shared DEGs and 3 shared pathways, representing 1% and 0.6% of the total in rabbits. Moreover, the number of opposite pathways triggered by tin perovskite (12 pathways) was four times higher than that observed for lead. Collectively, these findings indicate that, compared with lead perovskite, tin perovskite induces fewer shared DEGs and pathways but more opposite responses across species, accounting for its higher species sensitivity.

## 3 Conclusion

In summary, we report a unique toxicity comparison phenomenon—namely, the reversal of toxicity ranking between two perovskite compositions across different animal species. All toxicity indicators, including body weight, organ index, and blood biochemistry, were normalized to the corresponding reference samples and converted into dimensionless parameters, enabling direct and quantitative comparison of toxicity responses across different species and material compositions. Our results reveal that the toxicity variation induced by species change (from mice to rabbits) is significantly greater than that caused by compositional change (from lead to tin perovskite). The tiny overlap of damaged genes and biological pathways between mice and rabbits accounts for the pronounced interspecies toxicity differences. Moreover, tin‐based perovskites exhibit greater sensitivity to species variation than their lead‐based counterparts. Consequently, while lead‐based perovskites are more toxic in mice, this trend reverses in rabbits, where tin‐based perovskites show higher toxicity. This work provides new insights into how species‐dependent biomolecular responses fundamentally influence toxicity comparison outcomes.

In the present study, healthy female animals were employed for toxicity assessment. We would like to emphasize that physiological and metabolic processes in animals exhibit pronounced sex‐dependent differences; consequently, toxicological responses may display sexual dimorphism [37]. In addition to sex, other biological variables—including age, pregnancy status, and underlying disease conditions (e.g., tumor‐bearing or pre‐existing systemic disorders)—may also substantially influence toxicological outcomes. Therefore, more comprehensive investigations incorporating these variables are warranted to fully elucidate the safety profile.

## 4 Methods

The details of the experiments are as follows:

### 4.1 Ethics

All experiments involving animals were conducted in compliance with the regulations set forth by the China Committee for Research and Animal Ethics (SYXK 2022‐0015).

### 4.2 Mice

Female BALB/c mice aged 6–8 weeks were randomly divided into three groups of 8 mice each. Control: Mice were given DI water orally every day for two weeks. Lead perovskite group: Mice were given lead perovskite (MAPbI_3_) with a lead concentration of 20 mg/kg daily for two weeks. Tin perovskite group: Mice were given tin perovskite (MASnI_3_) with a tin concentration of 20 mg/kg daily for two weeks. After two weeks, the mice were weighed and sacrificed, and major organs (heart, kidney, liver, lung, spleen) and blood samples were collected for next characterization. For the low dose exposure experiment, mice were given lead or tin perovskite at a daily intake of 1 mg/kg or 0.5 mg/kg for two weeks.

### 4.3 Rabbits

Female New Zealand rabbits aged 14–16 weeks were randomly divided into three groups of 8 rabbits each. Control: Rabbits were given DI water orally every day for two weeks. Lead Perovskite Group: Rabbits were given lead perovskite (MAPbI_3_) with a lead concentration of 20 mg/kg daily for two weeks. Tin Perovskite Group: Rabbits were given tin perovskite (MASnI_3_) with a tin concentration of 20 mg/kg daily for two weeks. For the low dose exposure experiment, rabbits were given lead or tin perovskite with a daily intake of 1 mg/kg or 0.5 mg/kg for two weeks.

### 4.4 RNA Sequencing

The clearance of administered chemicals from the animal body is critical to suppressing the long‐lasting damage caused by perovskite, which is largely determined by kidney function. Thus, the kidney has been employed for the RNA sequencing analysis. In this text, all RNA sequencing data refer to the kidney organ.

Kidney tissues were homogenized with a tissue homogenizer. RNA was isolated from kidney tissue according to the vendor's protocol, and reverse transcription was conducted using the commercialized QuantiTect Reverse Transcription Kit (QIAGEN #205314). The integrity of the isolated RNA was assessed by means of an Agilent 2100 Bioanalyzer with the Agilent RNA 6000 Kit. Subsequently, RNA was reversely transcribed into cDNA, and libraries were established.

The RNA libraries were sequenced on a BGI platform. The resulting raw RNA sequencing data were aligned to either the rabbit genome reference OryCun2.0 or the mouse genome reference mm10 using Hisat2 (v2.1.0) with default parameters. FeatureCounts (v1.6.3) was employed to quantify the number of reads mapped to each gene. Differentially expressed genes (DEGs) were identified using DESeq2 (v1.34.0) and GO (Gene ontology) pathway enrichment analysis was performed by adopting clusterProfiler (v4.2.2).

### 4.5 RNA Isolation and Quantitative Real‐Time PCR

Total RNA was extracted from kidney tissues using the manufacturer's recommended protocol. RNA concentration and purity were determined spectrophotometrically (NanoDrop 2000, Thermo Fisher Scientific), and integrity was confirmed by agarose gel electrophoresis. Only samples with A260/280 ratios between 1.8 and 2.0 were used for downstream analysis. Reverse transcription was performed with the QuantiTect Reverse Transcription Kit (QIAGEN, #205314) according to the supplier's instructions, including genomic DNA elimination steps.

Quantitative real‐time PCR (qPCR) was carried out using SYBR Green ER Master Mix (Roche, #24759100) on a QuantStudio 7 Flex Real‐Time PCR System (Thermo Fisher Scientific). Each reaction was performed in triplicate with a final volume of 10 µL, containing 10 ng of cDNA and 0.2 µm of each primer. Melt curve analysis was performed to verify amplification specificity.

Relative gene expression levels were calculated using the 2^−ΔΔCt method, with 18s serving as the internal reference gene. Negative controls without a template were included in each run to exclude contamination.

### 4.6 Western Blot Analysis

Tissues were lysed in ice‐cold RIPA buffer (50 mm Tris‐HCl, pH 7.4; 150 mm NaCl; 1% NP‐40; 0.1% SDS; 0.5% sodium deoxycholate; 1 mm EDTA; and 10% glycerol) supplemented with protease and phosphatase inhibitor cocktails (Thermo Fisher Scientific). Lysates were incubated on ice for 30 min with intermittent vortexing and clarified by centrifugation at 12,000 × g for 15 min at 4°C. Protein concentrations were determined using a BCA Protein Assay Kit (Thermo Fisher Scientific), and equal amounts of protein were resolved by SDS‐PAGE on 10%–12% polyacrylamide gels.

Proteins were transferred onto PVDF membranes (Millipore) using a wet transfer system. Membranes were blocked with 5% non‐fat dry milk in Tris‐buffered saline containing 0.1% Tween‐20 (TBST) for 1 h at room temperature. Primary antibodies were incubated overnight at 4°C in blocking buffer, followed by incubation with appropriate secondary antibodies for 1 h at room temperature. Membranes were washed three times with TBST (10 min each) between incubations. Protein bands were visualized and quantified using the ODYSSEY CLx Imaging System. Band intensities were normalized to β‐actin as loading controls.

### 4.7 Statistical Analysis

One tailed distribution and two sample unequal variance Student's T test were utilized to evaluate the differences between two individual groups. p < 0.05 was regarded as statistically significant. ^*^p < 0.05, ^**^p ≤ 0.01, ^***^p ≤ 0.001, ^****^p ≤ 0.0001. The log_2_fold change >0 or <0 with p value <0.05 were employed as the filter threshold for up regulated and down regulated genes respectively.

## Conflicts of Interest

The authors declare no conflicts of interest.

## Supporting information




**Supporting File**: advs75102‐sup‐0001‐SuppMat.docx.

## Data Availability

The data that support the findings of this study are available from the corresponding author upon reasonable request.

## References

[1] Y. Shao , X. Hua , Y. Li , and D. Wang , “Comparison of Reproductive Toxicity between Pristine and Aged Polylactic Acid Microplastics in Caenorhabditis elegans,” Journal of Hazardous Materials 466 (2024): 133545, 10.1016/j.jhazmat.2024.133545. 38244453

[2] T. Schlender , M. Viljanen , J. N. van Rijn , et al., “The Bigger Fish: A Comparison of Meta‐Learning QSAR Models on Low‐Resourced Aquatic Toxicity Regression Tasks,” Environmental Science & Technology 57, no. 46 (2023): 17818–17830, 10.1021/acs.est.3c00334. 37315216 PMC10666535

[3] R. Dusautoir , G. Zarcone , M. Verriele , et al., “Comparison of the Chemical Composition of Aerosols from Heated Tobacco Products, Electronic Cigarettes and Tobacco Cigarettes and Their Toxic Impacts on the human Bronchial Epithelial BEAS‐2B Cells,” Journal of Hazardous Materials 401 (2021): 123417, 10.1016/j.jhazmat.2020.123417. 32763707

[4] W. Ding , Z. Li , R. Qi , et al., “Effect Thresholds for the Earthworm Eisenia Fetida: Toxicity Comparison between Conventional and Biodegradable Microplastics,” Science of The Total Environment 781 (2021): 146884, 10.1016/j.scitotenv.2021.146884.

[5] G. del Giudice , A. Serra , A. Pavel , et al., “A Network Toxicology Approach for Mechanistic Modelling of Nanomaterial Hazard and Adverse Outcomes,” Advanced Science 11, no. 32 (2024): 2400389, 10.1002/advs.202400389. 38923832 PMC11348149

[6] Z. Liu , C. Xie , T. Heumueller , et al., “A Review on Organic Nanoparticle‐based Optoelectronic Devices: From Synthesis to Applications,” Energy & Environmental Science 18, no. 1 (2025): 155–193, 10.1039/D4EE03575E.

[7] B. J. Curtis , N. J. Niemuth , E. Bennett , et al., “Cross‐species Transcriptomic Signatures Identify Mechanisms Related to Species Sensitivity and Common Responses to Nanomaterials,” Nature Nanotechnology 17, no. 6 (2022): 661–669, 10.1038/s41565-022-01096-2. 35393598

[8] G. del Giudice , A. Serra , L. A. Saarimäki , et al., “An Ancestral Molecular Response to Nanomaterial Particulates,” Nature Nanotechnology 18, no. 8 (2023): 957–966, 10.1038/s41565-023-01393-4. PMC10427433 37157020

[9] M. Tian , D. Wu , X. Gou , R. Li , and X. Zhang , “Genetic Modulation of Rare Earth Nanoparticle Biotransformation Shapes Biological Outcomes,” Nature Communications 16, no. 1 (2025): 3429, 10.1038/s41467-025-58520-z. PMC11985947 40210885

[10] B. Fadeel and A. A. Keller , “Nanosafety: A Perspective on Nano‐Bio Interactions,” Small 20, no. 28 (2024): 2310540, 10.1002/smll.202310540. 38597766

[11] A. Babayigit , D. Duy Thanh , A. Ethirajan , et al., “Assessing the Toxicity of Pb‐ and Sn‐based Perovskite Solar Cells in Model Organism Danio rerio,” Scientific Reports 6, no. 1 (2016): 18721, 10.1038/srep18721. 26759068 PMC4725943

[12] A. Babayigit , A. Ethirajan , M. Muller , and B. Conings , “Toxicity of Organometal Halide Perovskite Solar Cells,” Nature Materials 15, no. 3 (2016): 247–251, 10.1038/nmat4572. 26906955

[13] G. Li , Y.‐T. Liu , F. Yang , et al., “Biotoxicity of Halide Perovskites in Mice,” Advanced Materials 36, no. 2 (2024): 2306860, 10.1002/adma.202306860. 37703533

[14] N. Tagaras , H. Song , S. Sahar , W. Tong , Z. Mao , and T. Buerki‐Thurnherr , “Safety Landscape of Therapeutic Nanozymes and Future Research Directions,” Advanced Science 11, no. 46 (2024): 2407816, 10.1002/advs.202407816. 39445544 PMC11633477

[15] M. Faria , M. Björnmalm , K. J. Thurecht , et al., “Minimum Information Reporting in Bio–nano Experimental Literature,” Nature Nanotechnology 13, no. 9 (2018): 777–785, 10.1038/s41565-018-0246-4. PMC6150419 30190620

[16] The Risks of Nanomaterial Risk Assessment. Nature Nanotechnology 15, no. 3 (2020): 163–163, 10.1038/s41565-020-0658-9. 32157240

[17] B. Fadeel and K. Kostarelos , “Grouping all Carbon Nanotubes into a Single Substance Category Is Scientifically Unjustified,” Nature Nanotechnology 15, no. 3 (2020): 164–164, 10.1038/s41565-020-0654-0. 32123379

[18] A. Nel , T. Xia , L. Mädler , and N. Li , “Toxic Potential of Materials at the Nanolevel,” Science 311, no. 5761 (2006): 622–627, 10.1126/science.1114397. 16456071

[19] L. Xiao , T. An , C. Deng , X. Xu , and H. Sun , “On Biosafety of Sn‐containing Halide Perovskites,” Energy & Environmental Science 16, no. 5 (2023): 2120–2132, 10.1039/D2EE02510H.

[20] I. Maietta , C. Otero‐Martínez , S. Fernández , et al., “The Toxicity of Lead and Lead‐Free Perovskite Precursors and Nanocrystals to Human Cells and Aquatic Organisms,” Advanced Science 12, no. 13 (2025): 2415574, 10.1002/advs.202415574. 39927780 PMC11967815

[21] Q. Jiang , R. Tirawat , R. A. Kerner , et al., “Towards Linking Lab and Field Lifetimes of Perovskite Solar Cells,” Nature 623, no. 7986 (2023): 313–318, 10.1038/s41586-023-06610-7. 37696288

[22] Q. Li , D. Li , Z. Li , et al., “Tailoring Crystal Growth Regulation and Dual Passivation for Air‐Processed Efficient Perovskite Solar Cells,” Advanced Science 12, no. 14 (2025): 2407401, 10.1002/advs.202407401. 39973078 PMC11984867

[23] J. Park , S. Kim , W. Kim , Z. Sun , B. Lee , and C. Yang , “Stepwise Volatilization Induced by Nature‐sourced Volatile Solid Additives Improving the Efficiency and Stability of Perovskite Solar Cells,” Energy & Environmental Science 18, no. 12 (2025): 6260–6272, 10.1039/D4EE03897E.

[24] M. Li , Y. Yang , Z. Kuang , et al., “Acceleration of Radiative Recombination for Efficient Perovskite LEDs,” Nature 630, no. 8017 (2024): 631–635, 10.1038/s41586-024-07460-7. 38811739 PMC11186751

[25] Y. Guo , P. Yang , F. Dong , et al., “Lattice Stabilized and Emission Tunable Pure‐Bromide Quasi‐2D Perovskite for Air‐Processed Blue Light‐Emitting Diodes,” Advanced Science 12, no. 5 (2025): 2414499, 10.1002/advs.202414499. 39641384 PMC11791954

[26] C. Qin , A. S. D. Sandanayaka , C. Zhao , et al., “Stable Room‐temperature Continuous‐wave Lasing in Quasi‐2D Perovskite Films,” Nature 585, no. 7823 (2020): 53–57, 10.1038/s41586-020-2621-1. 32879501

[27] Z. Wang , X. Li , C. Chen , et al., “High Relative Humidity‐Induced Growth of Perovskite Nanowires from Glass toward Single‐Mode Photonic Nanolasers at Sub‐100‐Nm Scale,” Advanced Science 12, no. 5 (2025): 2412397, 10.1002/advs.202412397. 39665147 PMC11791984

[28] K. Sakhatskyi , B. Turedi , G. J. Matt , et al., “Stable Perovskite Single‐Crystal X‐Ray Imaging Detectors with Single‐Photon Sensitivity,” Nature Photonics 17, no. 6 (2023): 510–517, 10.1038/s41566-023-01207-y.

[29] J. Guo , C. Wang , X. Zhao , et al., “Synthesis of Perovskite Nanowires and Their Application for Photodetectors,” Advanced Science 12, no. 38 (2025): 10428, 10.1002/advs.202510428. PMC12520487 40847779

[30] H. Zhang , J.‐W. Lee , G. Nasti , et al., “Lead Immobilization for Environmentally Sustainable Perovskite Solar Cells,” Nature 617, no. 7962 (2023): 687–695, 10.1038/s41586-023-05938-4. 37225881

[31] R. Vidal , J.‐A. Alberola‐Borràs , S. N. Habisreutinger , et al., “Assessing Health and Environmental Impacts of Solvents for Producing Perovskite Solar Cells,” Nature Sustainability 4, no. 3 (2021): 277–285, 10.1038/s41893-020-00645-8.

[32] A. Abate , “Perovskite Solar Cells Go Lead Free,” Joule 1, no. 4 (2017): 659–664, 10.1016/j.joule.2017.09.007.

[33] J. Li , H.‐L. Cao , W.‐B. Jiao , et al., “Biological Impact of Lead from Halide Perovskites Reveals the Risk of Introducing a Safe Threshold,” Nature Communications 11, no. 1 (2020): 310, 10.1038/s41467-019-13910-y. PMC6974608 31964862

[34] F. De Angelis , “The Prospect of Lead‐Free Perovskite Photovoltaics,” ACS Energy Letters 6, no. 4 (2021): 1586–1587.

[35] T. Wu , X. Liu , X. Luo , et al., “Lead‐free Tin Perovskite Solar Cells,” Joule 5, no. 4 (2021): 863–886, 10.1016/j.joule.2021.03.001.

[36] Z. Xiao , Z. Song , and Y. Yan , “From Lead Halide Perovskites to Lead‐Free Metal Halide Perovskites and Perovskite Derivatives,” Advanced Materials 31, no. 47 (2019): 1803792, 10.1002/adma.201803792. 30680809

[37] M. Lei , Z. Zhu , C. Wei , et al., “Prenatal Silicon Dioxide Nanoparticles Exposure Reduces Female Offspring Fertility without Affecting Males,” Advanced Science 12, no. 3 (2025): 2410353, 10.1002/advs.202410353. 39574356 PMC11744561

