# Supplementary material for: Species Dependent Toxicity Comparison Outcome
Source: Adv Sci (Weinh). 2026 Mar 31;13(34):e23889. doi: 10.1002/advs.202523889 (PMC13285131; doi:10.1002/advs.202523889)
Supplement: Supplementary file 1 — Supporting File: advs75102‐sup‐0001‐SuppMat.docx. [file ADVS-13-e23889-s001.docx]

**Species Dependent Toxicity Comparison Outcome**

Lian Xiao^1, 2*^, Zhan Yu^3^, Sihang Liu^4^, Yuheng Liu^4^, Chong Deng^4^, Yugang Zhao^5^, Yi Huang *^4^, Zhi-Gang Zheng^1^*

*Emails: [xiaolian@ecust.edu.scn](mailto:xiaolian@ecust.edu.scn), [huangyi_buaa@buaa.edu.cn](mailto:huangyi_buaa@buaa.edu.cn), zgzheng@ecust.edu.cn

^1^ School of Physics, East China University of Science and Technology, Shanghai 200237, China

^2^ Division of Physics and Applied Physics, School of Physical and Mathematical Sciences, Nanyang Technological University, 21 Nanyang Link, Singapore 637371.

^3^ Beijing An Zhen Hospital, Affiliated of Capital University of Medical Sciences

^4^ Research Institute of Aero-Engine, Beihang University, No.37 XueYuan Road, Haidian District, Beijing, China, 100083

^5^ Shanghai Key Laboratory of Multiphase Flow and Heat Transfer in Power Engineering, School of Energy and Power Engineering, University of Shanghai for Science and Technology, Shanghai 200093, China

**Keywords**: toxicity comparison; toxicity mechanism; toxicity evaluation; biosafe; perovskite

**Experimental design:**

Mice and rabbits were utilized for the experiments for the following reasons: 1. Both are mammals, allowing for same exposure conditions and feeding strategies. 2. Both can be orally administered perovskite, which not only mimics human perovskite intake but also allows for precise control of the perovskite intake amount. These properties enable precise control and quantification of exposure conditions, minimizing confounding factors, and thus identifying the true toxicity mechanism. The approach for perovskite intake was based on previously utilized methods with slightly modification^1-3^. Specifically, perovskite (MASnI_3_: SnI_2_ + MAI (Methylammonium iodide); MAPbI_3_: PbI_2_ + MAI (Methylammonium iodide)) were dissolved in water and orally administered to the animals. Since the lead and tin are the key element in perovskite, we employ the lead and tin concentration to represent the perovskite concentration.


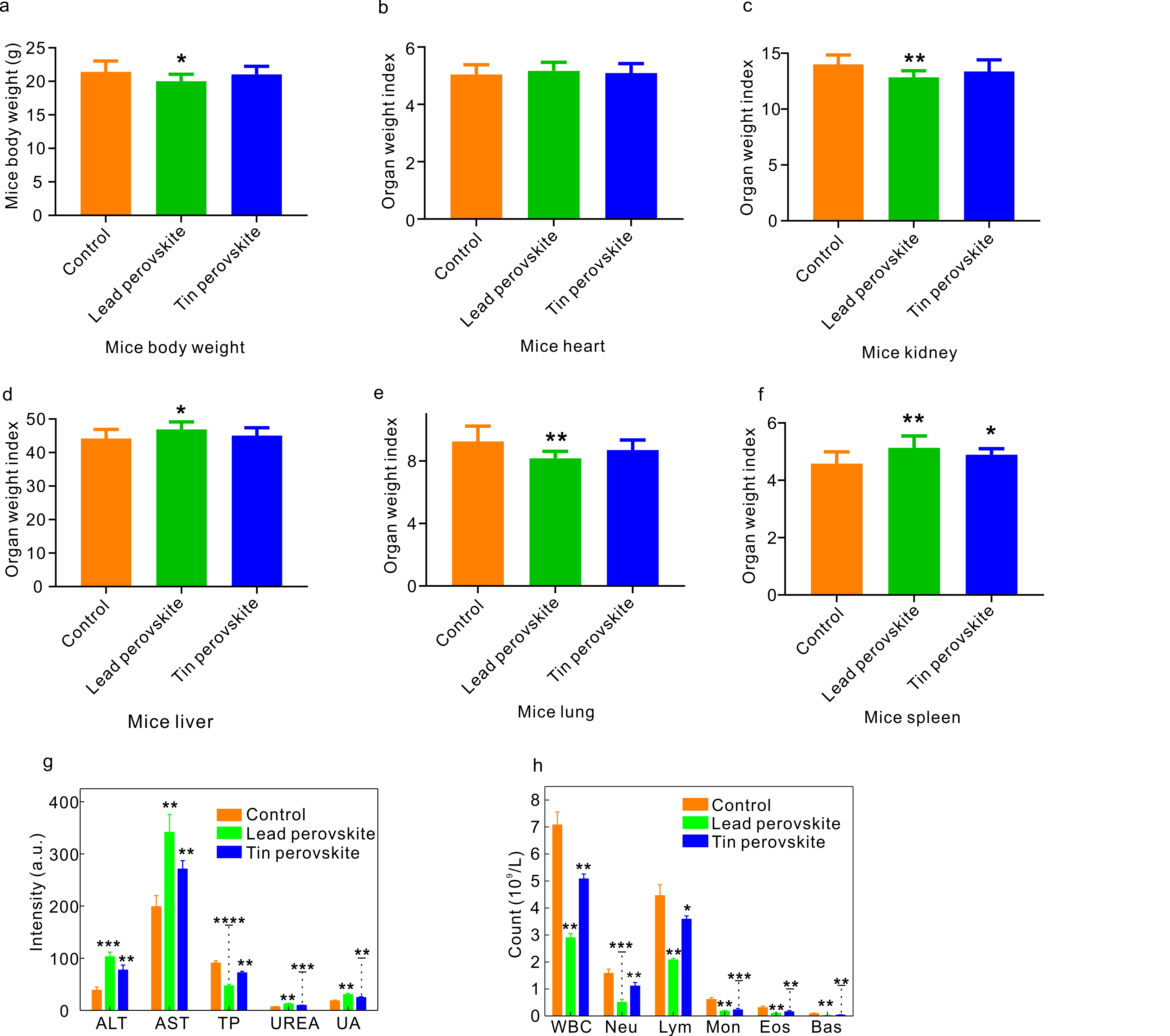


**Figure S1** Original data of toxicity evaluation of lead and tin halide perovskite in mice. (a) Mice body weight, (b) – (f) organ weight index for mice heart, kidney, liver, lung, and spleen, in control, lead perovskite, and tin perovskite treatment group, respectively. (g) Blood biochemistry measurement, Liver indicators: ALT, AST, TP. Kidney indicator: UREA, UA. (h) Hematology evaluation. Blood immune cells: WBC (white blood cells), Neu (neutrophils), Lym (lymphocytes), Mon (monocytes), Eos (eosinophils), Bas (basophils). For plots (a – f): n=8, error bars show mean ± SEM; for plots (g– h): n=3 or 4, error bars show mean ± SEM. The one side Student’s T-test was used to calculate significance. *P < 0.05, **P ≤ 0.01, ***P ≤0.001, ****P ≤ 0.0001. Daily intake: 20 mg/kg of lead from lead halide perovskite, or 20 mg/kg of tin from tin halide perovskite.


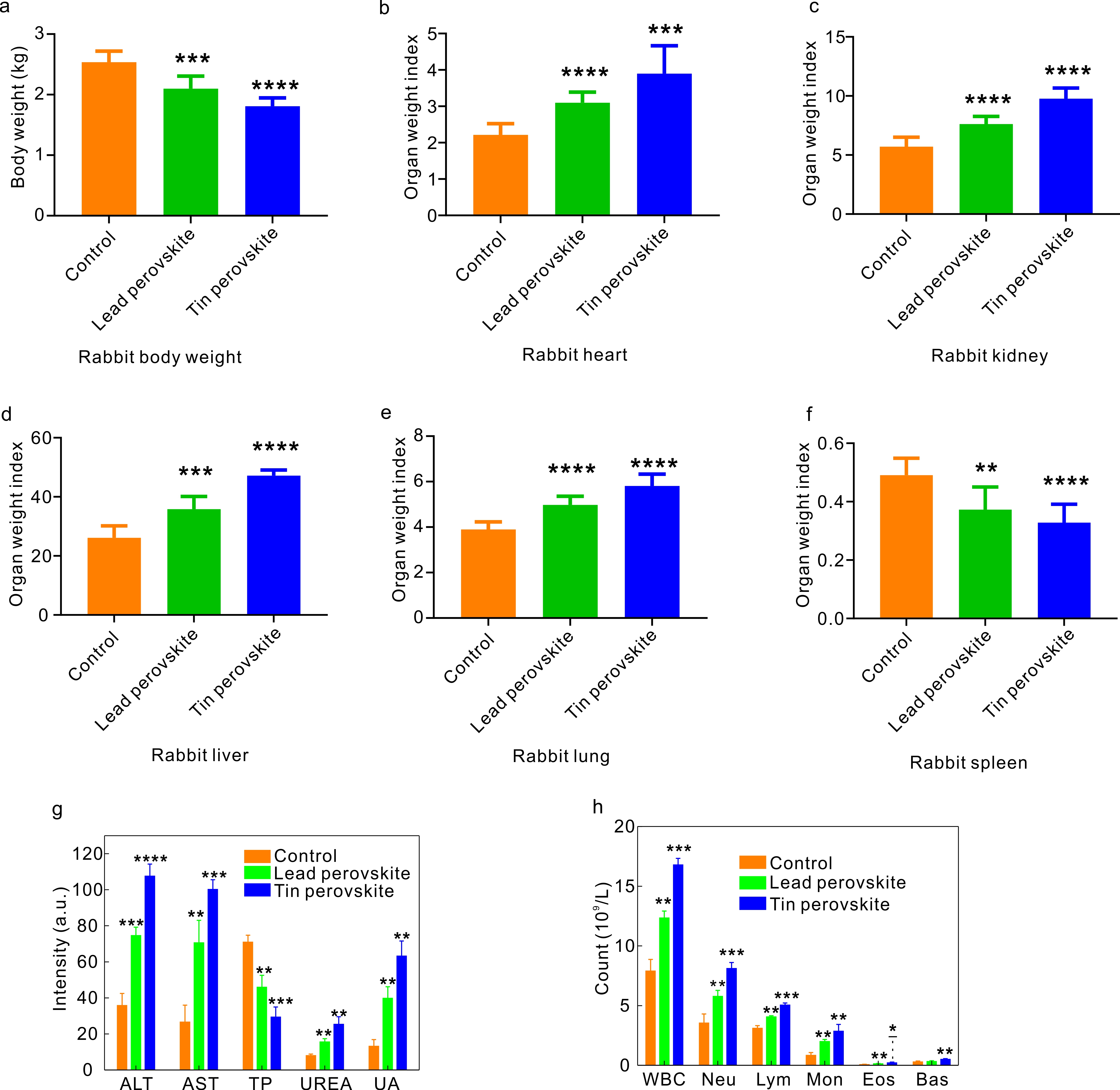


**Figure S2** Original data of toxicity evaluation of lead and tin halide perovskite in rabbit. (a) Rabbit body weight, (b) – (f) organ weight index for rabbit heart, kidney, liver, lung, and spleen, in control, lead perovskite, and tin perovskite treatment group, respectively. (g) Blood biochemistry measurement, Liver indicators: ALT, AST, TP. Kidney indicator: UREA, UA. (h) Blood immune cells: WBC (white blood cells), Neu (neutrophils), Lym (lymphocytes), Mon (monocytes), Eos (eosinophils), Bas (basophils). For plots (a – f): n=8, error bars show mean ± SEM; for plots (g– h): n=3 or 4, error bars show mean ± SEM. The one side Student’s T-test was used to calculate significance. *P < 0.05, **P ≤ 0.01, ***P ≤0.001, ****P ≤ 0.0001. Daily intake: 20 mg/kg of lead from lead halide perovskite, or 20 mg/kg of tin from tin halide perovskite.


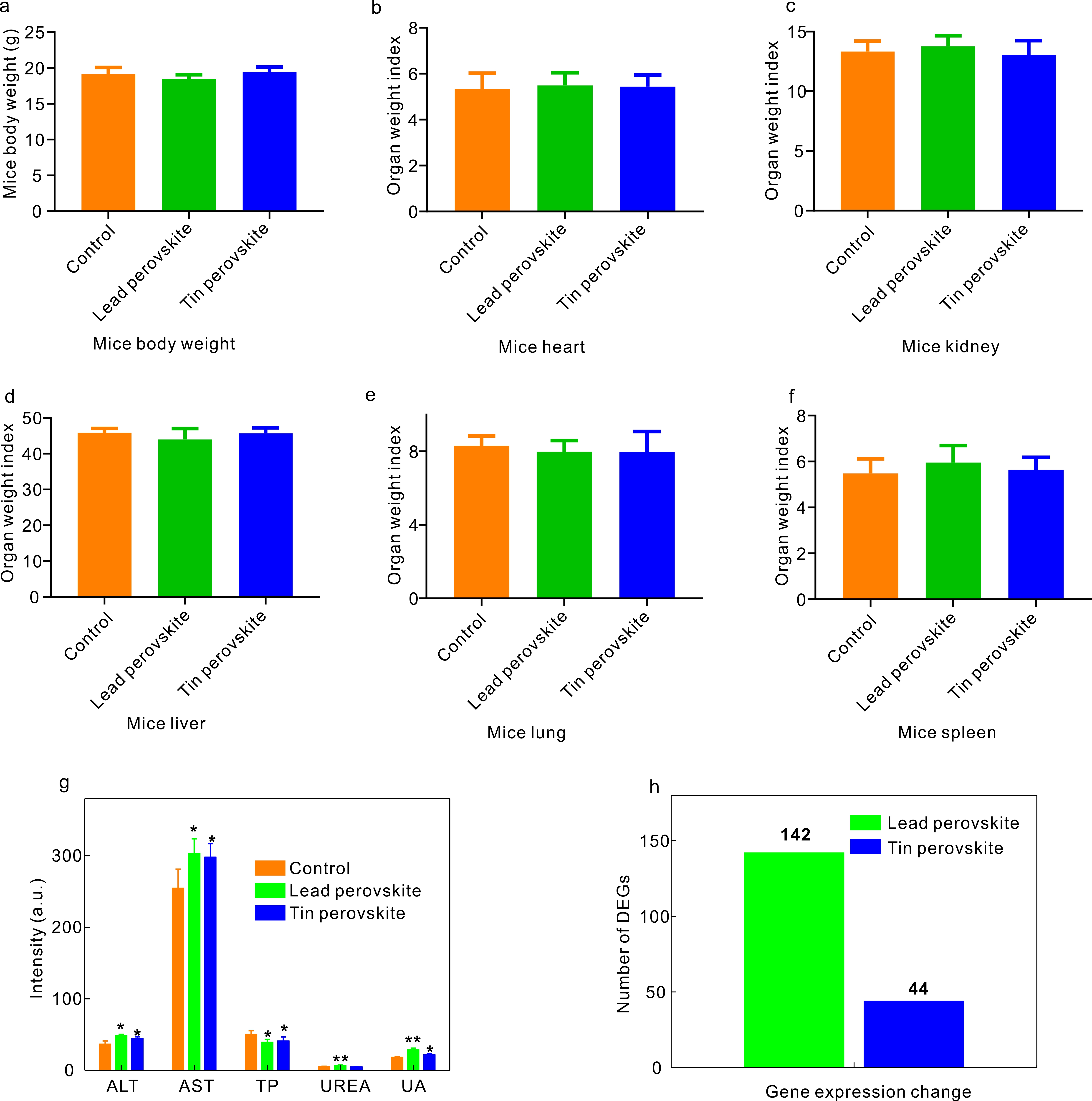


**Figure S3** Toxicity evaluation of lead and tin halide perovskite in mice. (a) Mice body weight, (b) – (f) organ weight index for mice heart, kidney, liver, lung, and spleen, in control, lead perovskite, and tin perovskite treatment group, respectively. (g) Blood biochemistry measurement, Liver indicators: ALT, AST, TP. Kidney indicator: UREA, UA. (h) The number of differentially expressed genes (DEGs) in lead and tin perovskite treatment groups. For plots (a – f): n=8, error bars show mean ± SEM; for plots (g): n=3 or 4, error bars show mean ± SEM. The one side Student’s T-test was used to calculate significance. *P < 0.05, **P ≤ 0.01, ***P ≤0.001, ****P ≤ 0.0001. Daily intake (orally): 1 mg/kg of lead from lead halide perovskite, or 1 mg/kg of tin from tin halide perovskite.


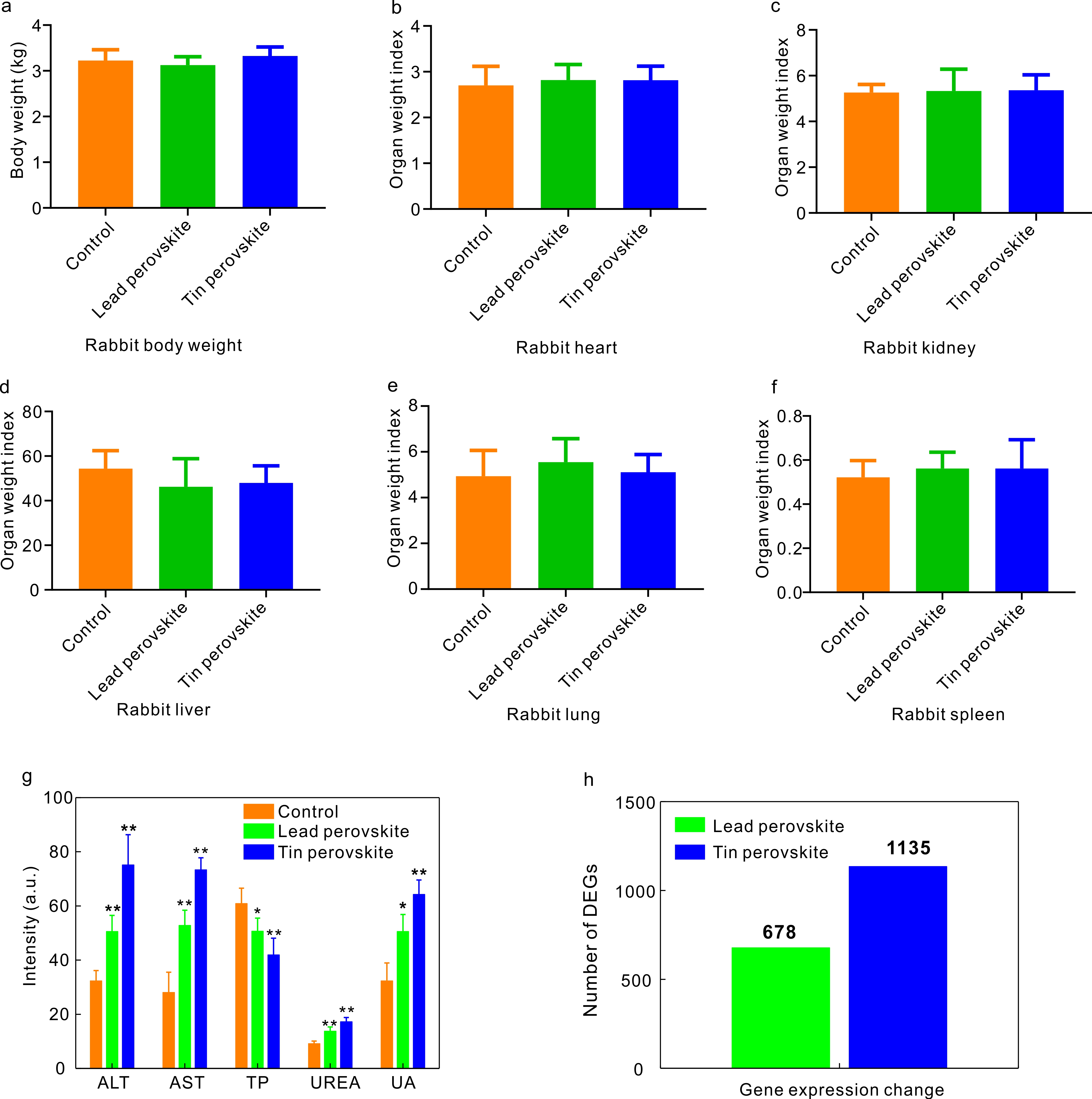


**Figure S4** Toxicity evaluation of lead and tin halide perovskite in rabbit. (a) Rabbit body weight, (b) – (f) organ weight index for rabbit heart, kidney, liver, lung, and spleen, in control, lead perovskite, and tin perovskite treatment group, respectively. (g) Blood biochemistry measurement, Liver indicators: ALT, AST, TP. Kidney indicator: UREA, UA. (h) The number of differentially expressed genes (DEGs) in lead and tin perovskite treatment groups. For plots (a – f): n=8, error bars show mean ± SEM; for plots (g): n=3 or 4, error bars show mean ± SEM. The one side Student’s T-test was used to calculate significance. *P < 0.05, **P ≤ 0.01, ***P ≤0.001, ****P ≤ 0.0001. Daily intake (orally): 1 mg/kg of lead from lead halide perovskite, or 1 mg/kg of tin from tin halide perovskite.


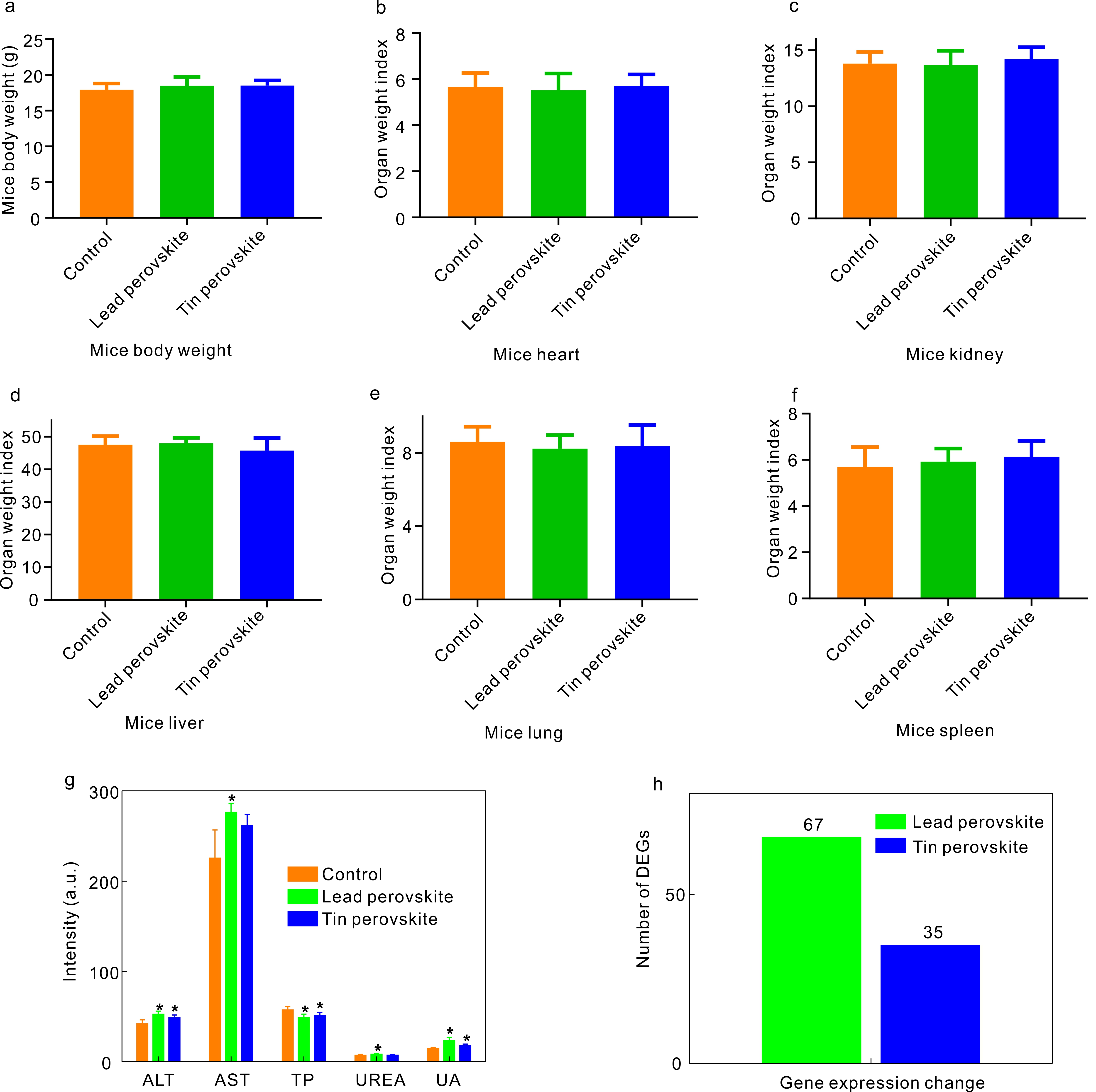


**Figure S5** Toxicity evaluation of lead and tin halide perovskite in mice. (a) Mice body weight, (b) – (f) organ weight index for mice heart, kidney, liver, lung, and spleen, in control, lead perovskite, and tin perovskite treatment group, respectively. (g) Blood biochemistry measurement, Liver indicators: ALT, AST, TP. Kidney indicator: UREA, UA. (h) The number of differentially expressed genes (DEGs) in lead and tin perovskite treatment groups. For plots (a – f): n=8, error bars show mean ± SEM; for plots (g): n=3 or 4, error bars show mean ± SEM. The one side Student’s T-test was used to calculate significance. *P < 0.05, **P ≤ 0.01, ***P ≤0.001, ****P ≤ 0.0001. Daily intake (orally): 0.5 mg/kg of lead from lead halide perovskite, or 0.5 mg/kg of tin from tin halide perovskite.


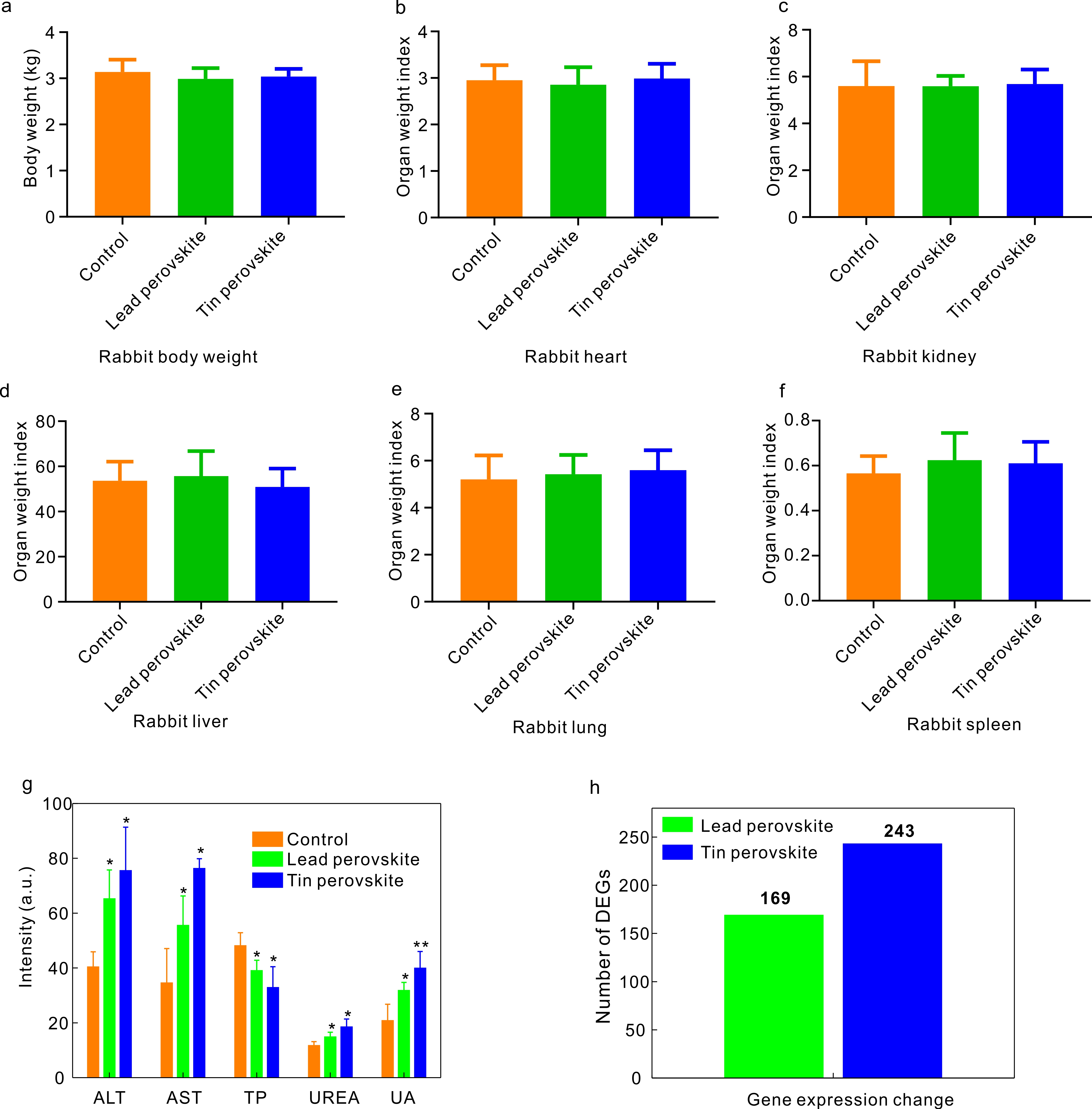


**Figure S6** Toxicity evaluation of lead and tin halide perovskite in rabbit. (a) Rabbit body weight, (b) – (f) organ weight index for rabbit heart, kidney, liver, lung, and spleen, in control, lead perovskite, and tin perovskite treatment group, respectively. (g) Blood biochemistry measurement, Liver indicators: ALT, AST, TP. Kidney indicator: UREA, UA. (h) The number of differentially expressed genes (DEGs) in lead and tin perovskite treatment groups. For plots (a – f): n=8, error bars show mean ± SEM; for plots (g): n=3 or 4, error bars show mean ± SEM. The one side Student’s T-test was used to calculate significance. *P < 0.05, **P ≤ 0.01, ***P ≤0.001, ****P ≤ 0.0001. Daily intake (orally): 0.5 mg/kg of lead from lead halide perovskite, or 0.5 mg/kg of tin from tin halide perovskite.


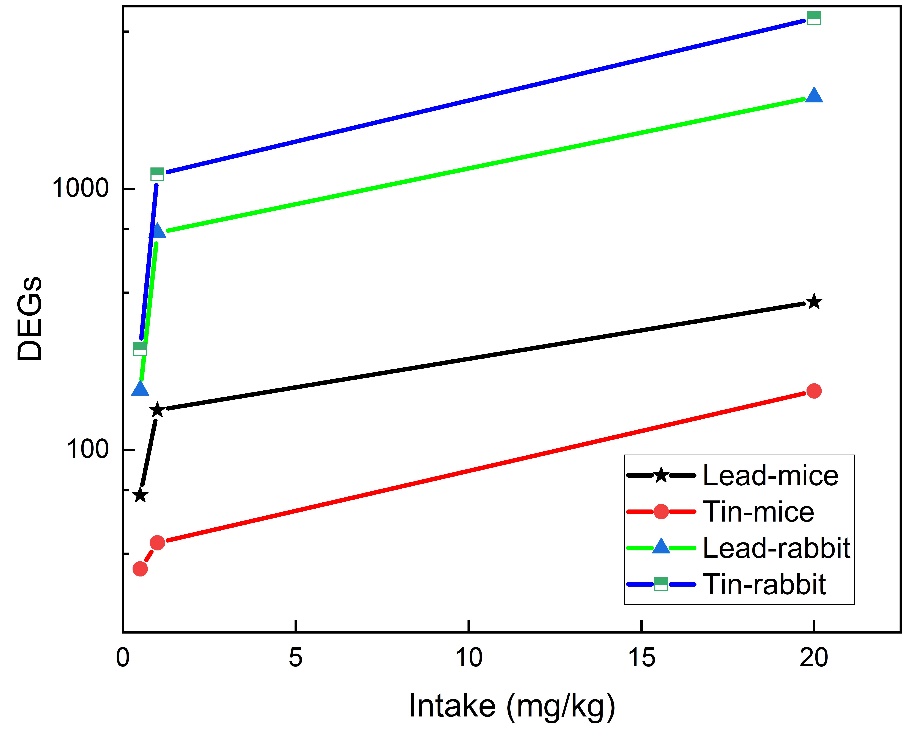


**Figure S7** DEGs as a function of intake dose (kidney tissue).

For protein expression analysis, validation was conducted in mice only. Because rabbits are not a commonly used model for this type of molecular analysis, commercially available antibodies validated for rabbit proteins are limited, and suitable antibodies could not be identified from major suppliers.

The protein expression levels of the leading-edge (core enrichment) genes from the Top 10 significantly altered pathways—selected based on both log_2_FC and FDR—are shown below. These protein-level results are consistent with the RNA-seq data, further validating the reliability of the transcriptomic analysis.

Lead perovskite exposure in mice


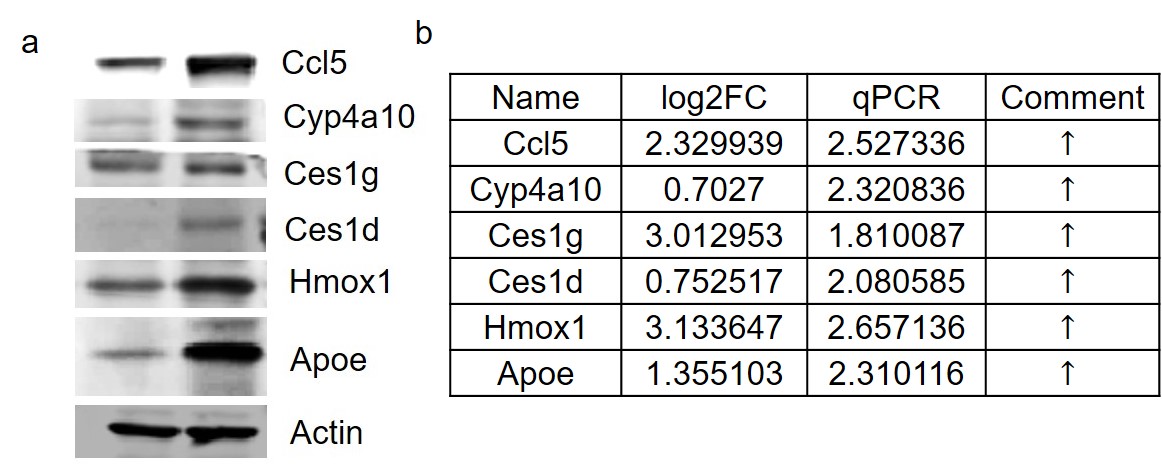


**Figure S8** (a) Western blot (WB) results of the key genes. (b) Expression levels of key genes identified by RNA-seq analysis and qPCR results. Lead perovskite exposure in mice.

Tin perovskite exposure in mice


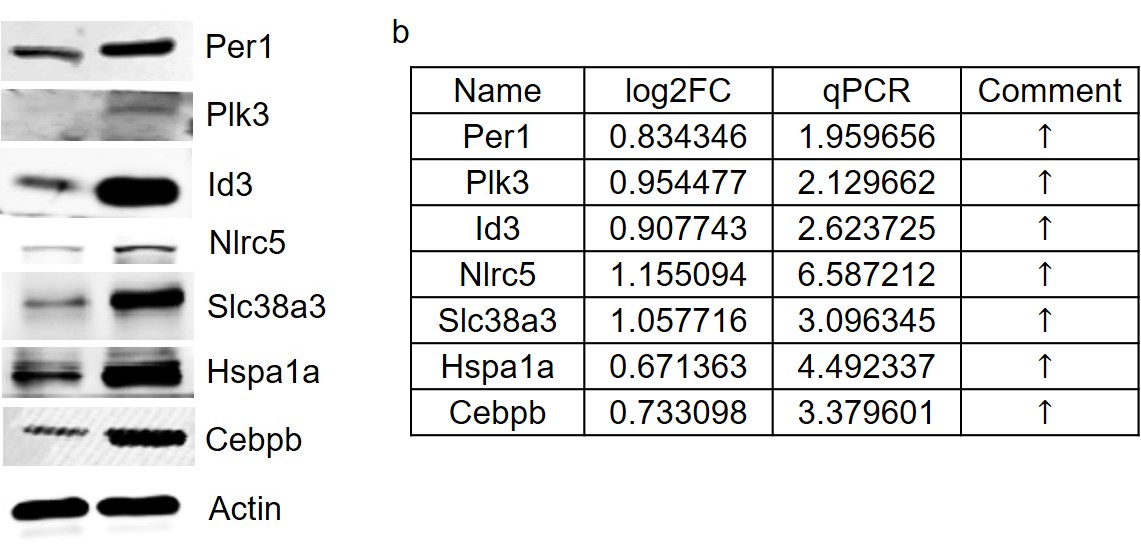


**Figure S9** (a) Western blot (WB) results of the key genes. (b) Expression levels of key genes identified by RNA-seq analysis and qPCR results. Tin perovskite exposure in mice.

Lead perovskite exposure in rabbit

**Table S1** Expression levels of key genes identified by RNA-seq analysis and qPCR results. Lead perovskite exposure in rabbit.

| ID | log2FC | QPCR results | Comment |
| --- | --- | --- | --- |
| NEK2 | 2.955113 | 4.103316 | $\uparrow$ |
| CDCA8 | 2.855967 | 3.347056 | $\uparrow$ |
| NCAPG | 1.254798 | 5.182883 | $\uparrow$ |
| KIF2C | 2.421957 | 2.088382 | $\uparrow$ |
| SGO1 | 2.52737 | 2.801011 | $\uparrow$ |
| NDC80 | 2.044966 | 3.035336 | $\uparrow$ |
| DMC1 | 5.008964 | 2.168961 | $\uparrow$ |
| RAD51 | 2.040577 | 1.987691 | $\uparrow$ |
| CDC6 | 1.567034 | 3.1082 | $\uparrow$ |
| BRCA1 | 1.423175 | 2.310455 | $\uparrow$ |
| ZWILCH | 2.304092 | 3.450785 | $\uparrow$ |
| KNL1 | 3.025553 | 6.966064 | $\uparrow$ |

Tin perovskite exposure in rabbit

**Table S2** Expression levels of key genes identified by RNA-seq analysis and qPCR results. Tin perovskite exposure in rabbit.

| ID | log2FC | QPCR results | Comment |
| --- | --- | --- | --- |
| ABCD3 | -1.74 | 0.517709 | $\downarrow$ |
| ABCB11 | -1.37 | 0.198373 | $\downarrow$ |
| EHHADH | -2.16 | 0.555112 | $\downarrow$ |
| ETFDH | -1.47 | 0.175444 | $\downarrow$ |
| HADHA | -1.29 | 0.446107 | $\downarrow$ |
| SCP2 | -1.29 | 0.362884 | $\downarrow$ |
| BDH2 | -1.13 | 0.172385 | $\downarrow$ |
| ADIPOQ | -2 | 0.634262 | $\downarrow$ |
| PEX13 | -1.07 | 0.652471 | $\downarrow$ |
| CHRNA5 | -5.62 | 0.218246 | $\downarrow$ |


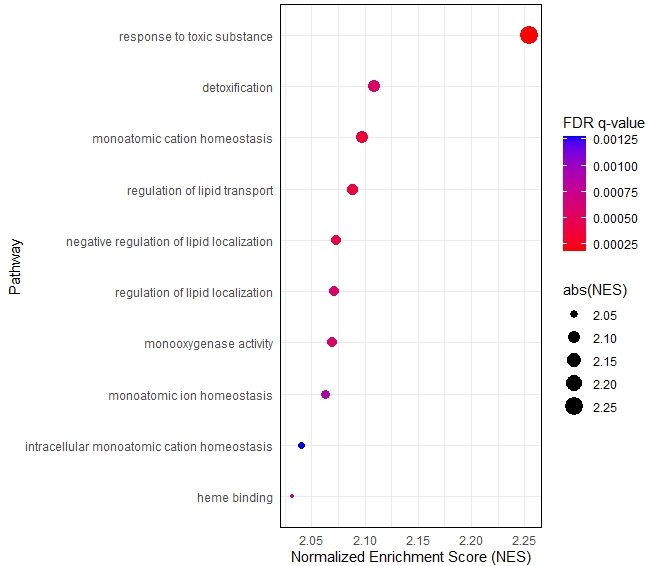


**Figure S10** Top 10 altered (impaired) physiological functions (pathways) for lead perovskite exposure in mice (kidney tissue). Daily intake: 20 mg/kg of lead from lead halide perovskite.


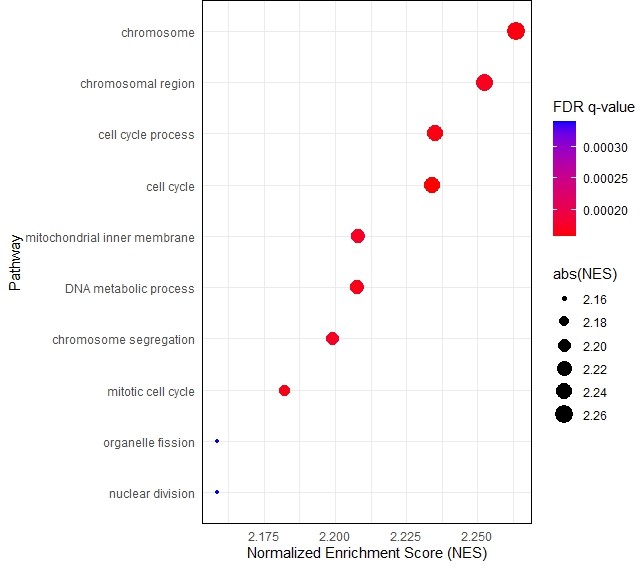


**Figure S11** Top 10 altered (impaired) physiological functions (pathways) for lead perovskite exposure in rabbit (kidney tissue). Daily intake: 20 mg/kg of lead from lead halide perovskite.


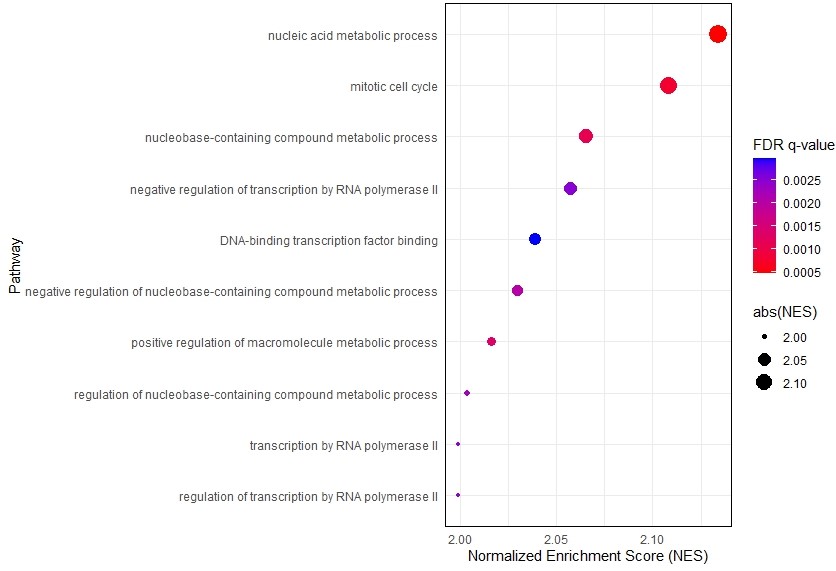


**Figure S12** Top 10 altered (impaired) physiological functions (pathways) for tin perovskite exposure in mice (kidney tissue). Daily intake: 20 mg/kg of tin from tin halide perovskite.


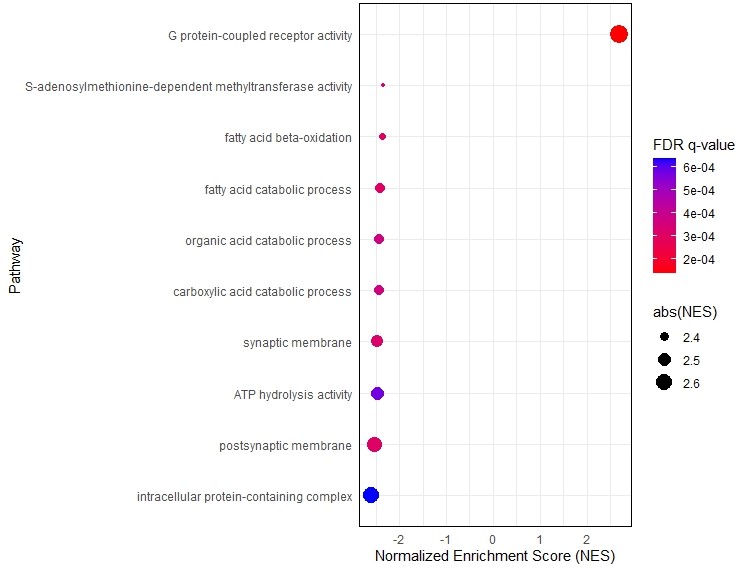


**Figure S13** Top 10 altered (impaired) physiological functions (pathways) for tin perovskite exposure in rabbit (kidney tissue). Daily intake: 20 mg/kg of tin from tin halide perovskite.


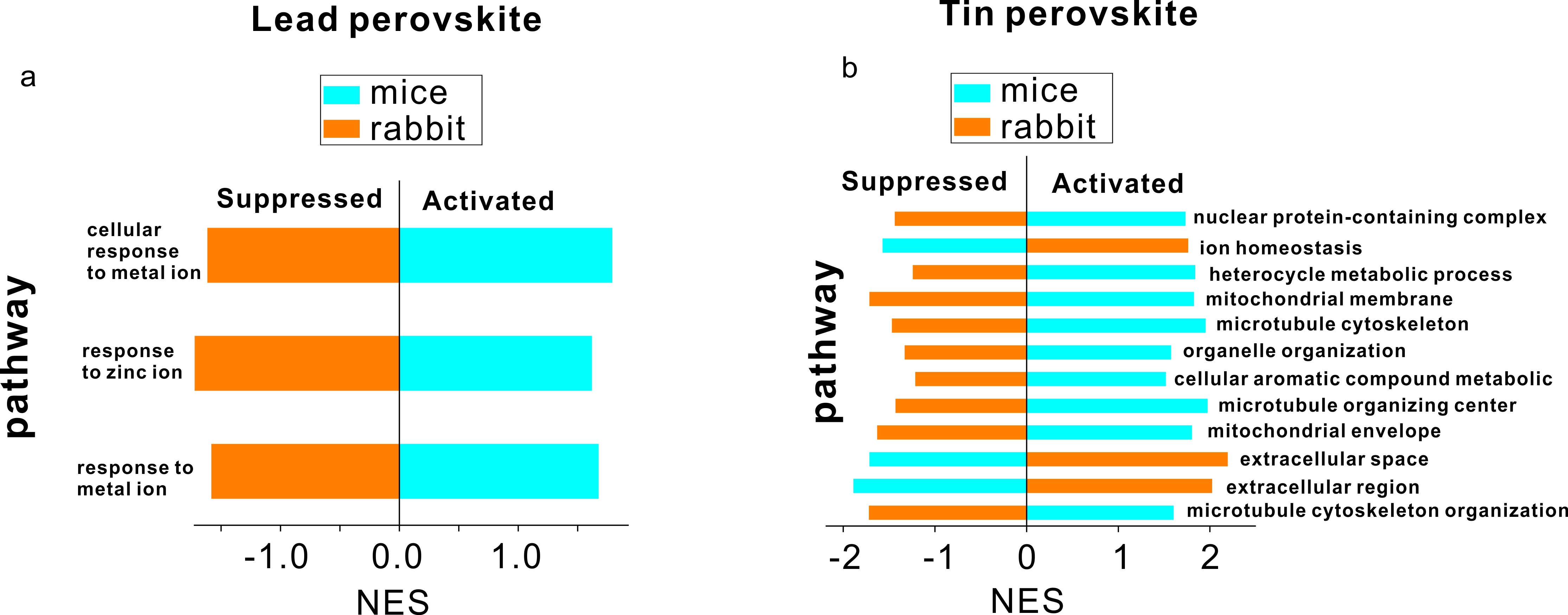


**Figure S14** (a) Opposing response physiological functions (pathways) between mice and rabbit for lead perovskite (kidney tissue). (b) Opposing response physiological functions (pathways) between mice and rabbit for tin perovskite (kidney tissue). Daily intake: 20 mg/kg of lead from lead halide perovskite, or 20 mg/kg of tin from tin halide perovskite.

**Primers utilized in this work**

| **Mouse** | Forward | Reverse |
| --- | --- | --- |
| **Plk3** | ATGGCCCTGTCAGCCAATAG | GCAAAATACCGCAGGATGCC |
| **Id3** | TGAGCTCACTCCGGAACTTG | GCAGGATTTCCCATGCAAGC |
| **Nlrc5** | AGACATTGCCCCTCTGTTCC | GCTGTTTCGGCTCAGGTCAA |
| **Per1** | ATGACATACCAGGTGCCGTC | GTCCTCTGAGAACCGTGGC |
| **Slc38a3** | CACCCTTCATCCCCTGCTG | GCCTCCTGTGTCTGTCTTCC |
| **Hspa1a** | GCTGAGGCTGTTGAGGAGTT | TCTTCTTGGGGTCCTTGTTG |
| **Cebpb** | GGGCTGCTTCTACTACGAGG | AGCTGCTTGAAGTTGCTGTT |
| **Apoe** | GCTGGGATTACCTGGAACAG | AGGCTGCTTCTGCAGGTAGT |
| **Hmox1** | GGAAATCATCCCTTGCACGC | TGTTTGAACTTGGTGGGGCT |
| **Ces1d** | GGCCAACTTTGCTCGGAATG | TTAAGCACAGCTGACCCTCC |
| **Ces1g** | GGCCAACTTTGCTCGGAATG | TCAAACATGACTGGGCCTCC |
| **Cyp4a10** | TGTACCATGGTCTCCAAAATCCA | GATGGAGAAACTCGTGTGAGG |
| **Ccl5** | GTGCTCCAATCTTGCAGTCG | CCCATTTTCCCAGGACCGAG |

| **Rabbit** | Forward | Reverse |
| --- | --- | --- |
| **ABCD3** | AGTGGAAAACCACCATTACAGA | CGAGACACCAGCATAACAGC |
| **ABCB11** | TGGGATTTTCCCAGTGGTGG | AAGCAGCCACTGTTCGCATA |
| **EHHADH** | TCGGCGCCCGGAAAAG | AGAAATTGCCACCTGCTCCA |
| **ETFDH** | GTTCCTTCGAACATGCTGGC | GGCGAACCTCTCCATGTTCA |
| **HADHA** | GGCATTGCTCAGGTCTCTGT | GGTAATCTAGCTGGCCCGTC |
| **BDH2** | AGGGAATCCGATGCAACTGT | GATGACCACGGGGTTACCTG |
| **ADIPOQ** | AGCTCCACTTGGCTTTCCA | TGCAGCAACAGCATCCTGAG |
| **PEX13** | GCGCTAGGTGCTTGTCTACC | GGAAGAATAGGTGGGGGCAC |
| **CHRNA5** | AGATCAAGTTTGGCCTCGCA | CAAGACGATGTCTGGGGTCC |
| **SCP2** | TTCAAGGTGAAGGATGGCCC | GCAATGGTGATCGTGCAGTC |
| **NEK2** | GAGCTCTGTGTCCGTGAGAG | TCCTCACTCCTCCCGATGTT |
| **CDCA8** | TGAGCCGATTGGAGTTGTCC | CTGTGCAATGTCGACCCTCT |
| **NCAPG** | TGACTCCTCGCAGGGATGTA | GCTGTTTTGGCTCGTCTTGG |
| **KIF2C** | CTGTTTCCCGGTCTCACCAT | CTGAGACAGTGGACATGCGA |
| **SGO1** | CGGGATGACAGGATTGTGCT | ATCTGGCATGGTGCAACTGT |
| **NDC80** | AGCGGAGTGTCACACAGTTT | TTGTCTGTTGAGGTCCTGCG |
| **DMC1** | AAAAACCCATCGGGGGACAC | TCACCACCTACTCCTTGGCA |
| **RAD51** | CCTGAAGCTGAGGCCATGTT | AGAATCCCCCAAGGAGCAGT |
| **CDC6** | GGCGGTGTGTGAGGGA | CGGAGTGTTGCATAGGTTGTC |
| **BRCA1** | AGGAAACCAGTCTTAGTGTCCA | TGCAGGATCCAAAGTGGCTT |
| **ZWILCH** | GTTCTCTAGGCCAGCGACTC | ATGTCACTGTAGCAGTCCGC |
| **KNL1** | GCATCAGCTAGGCGGTTAGA | AGAGGACTCCTTGGGGGTTT |

**References**

1 Li, J. *et al.* Biological impact of lead from halide perovskites reveals the risk of introducing a safe threshold. *Nature Communications* **11**, 310, doi:10.1038/s41467-019-13910-y (2020).

2 Li, G. *et al.* Biotoxicity of Halide Perovskites in Mice. *Advanced Materials* **36**, 2306860, doi:<https://doi.org/10.1002/adma.202306860> (2024).

3 Xiao, L., An, T., Deng, C., Xu, X. & Sun, H. On biosafety of Sn-containing halide perovskites. *Energy & Environmental Science* **16**, 2120-2132, doi:10.1039/D2EE02510H (2023).
